# Hyperpatulones A–F, polycyclic polyprenylated acylphloroglucinols from *Hypericum patulum* and their cytotoxic activities†

**DOI:** 10.1039/c9ra00277d

**Published:** 2019-03-12

**Authors:** Zhong-Nan Wu, Qian-Wen Niu, Yu-Bo Zhang, Ding Luo, Qing-Guo Li, Ying-Ying Li, Guang-Kai Kuang, Li-Jun He, Guo-Cai Wang, Yao-Lan Li

**Affiliations:** Institute of Traditional Chinese Medicine & Natural Products, Guangdong Province Key Laboratory of Pharmacodynamic Constituents of TCM and New Drugs Research, College of Pharmacy, Jinan University Guangzhou 510632 People's Republic of China twangguocai@jnu.edu.cn tliyl@jnu.edu.cn; Integrated Chinese and Western Medicine Postdoctoral Research Station, Jinan University Guangzhou 510632 People's Republic of China; School of Pharmaceutical Sciences, Guangzhou University of Chinese Medicine Guangzhou 510006 China

## Abstract

Six new compounds, hyperpatulones A–F (1–6), along with ten additional known related derivatives (7–16), were isolated from *Hypericum patulum* (Guttiferae). Their structures were elucidated by extensive analysis of spectroscopic data (IR, UV, HRESIMS, 1D and 2D NMR), X-ray crystallography, electronic circular dichroism (ECD) spectroscopy and Rh_2_(OCOCF_3_)_4_-induced ECD. All compounds were tested for their cytotoxic activities on human HepG-2, HeLa, MCF-7, and A549 cell lines *via* 3-(4,5-dimethylthiazol-2-yl)-2,5-diphenyltetrazolium bromide (MTT) assay. Compound 5 exhibited significant cytotoxicities against HepG-2, HeLa and A549 cell lines with IC_50_ values of 9.52 ± 0.27, 11.87 ± 0.22 and 12.63 ± 0.12 μM, respectively.

## Abstract 2

Six new PPAPs (1–6) were isolated from *Hypericum patulum* and compound 5 exhibited significant cytotoxicities on various tumor cell lines.

## Graphical abstract

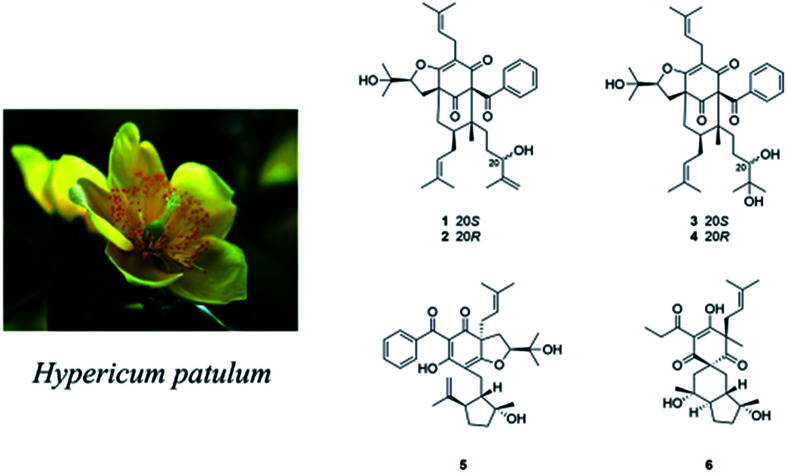

## Introduction

*Hypericum patulum* (Guttiferae) is well known as “Jinsimei” in China, and is distributed mainly in southwest China, such as Guizhou, Sichuan and Yunnan Provinces.^1^ The herbs of *H. patulum* are used as a traditional medicine to clear heat, cool blood, relax tendons and activate collaterals, and to treat gonorrhea, hepatitis, colds, *etc.*^2–6^ Modern pharmacological investigations demonstrated that the plants of the genus *Hypericum* possessed anti-depression,^7–11^ anti-tumor,^12–18^ anti-bacterial,^19,20^ anti-viral,^17,18,21–23^ and liver protective activities.^24^ Previous phytochemical studies on these plants showed that derivatives of polycyclic polyprenylated acylphloroglucinols (PPAPs), which possessed a highly oxygenated bicyclo[3.3.1]nonane-2,4,9-trione or other related core decorated with C_5_H_9_ or C_10_H_17_ (prenyl or geranyl) side chains, were the main bioactive components.^7,13–19,21,24,25^

**Fig. 1:**
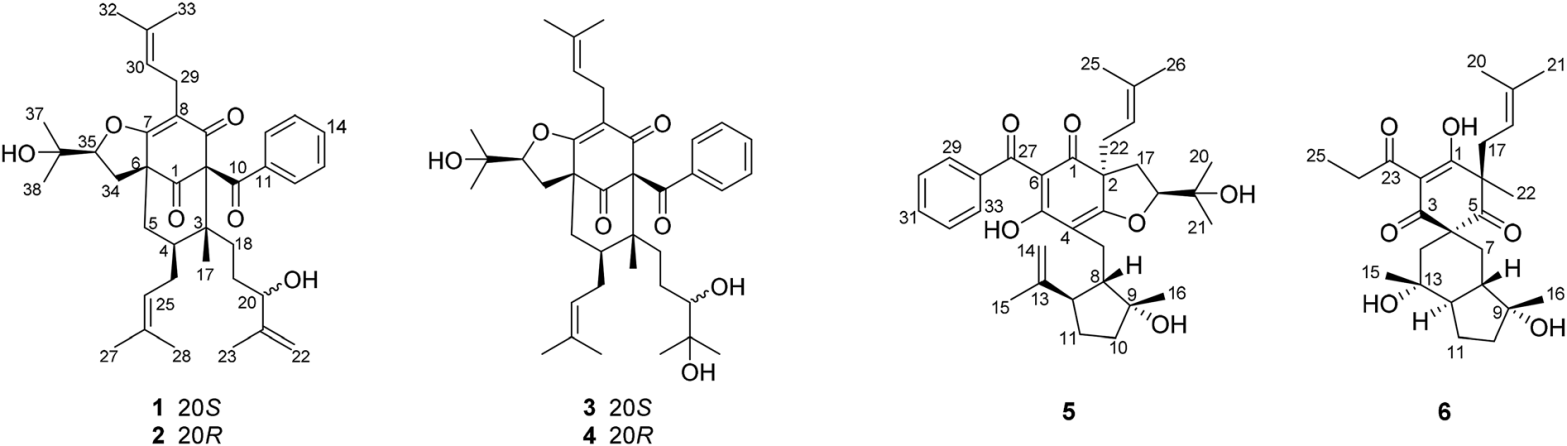
Chemical structures of 1–6.

In this paper, we report the isolation and structural elucidation of six new PPAPs (1–6) (Fig. 1), together with ten known ones (7–16). Their structures were elucidated using spectroscopic data, X-ray crystallography, ECD spectroscopy and Rh_2_(OCOCF_3_)_4_-induced ECD. Moreover, compounds 1–16 were evaluated for their cytotoxic activities on human HepG-2, HeLa, MCF-7, and A549 cell lines using the MTT assay. Among them, compound 5 shows significant cytotoxicities toward HepG-2, HeLa and A549 cell lines (IC_50_ = 9.52 ± 0.27, 11.87 ± 0.22 and 12.63 ± 0.12 μM).

## Results and discussion

The 95% EtOH extract of *Hypericum patulum* was subjected to liquid–liquid fractionation to afford a petroleum ether (PE)-soluble fraction and an ethyl acetate (EtOAc)-soluble fraction. The PE fraction was separated by silica gel column chromatography, Sephadex LH-20 and preparative HPLC to obtain six new compounds (1–6) and ten known ones (7–16).

**Fig. 2:**
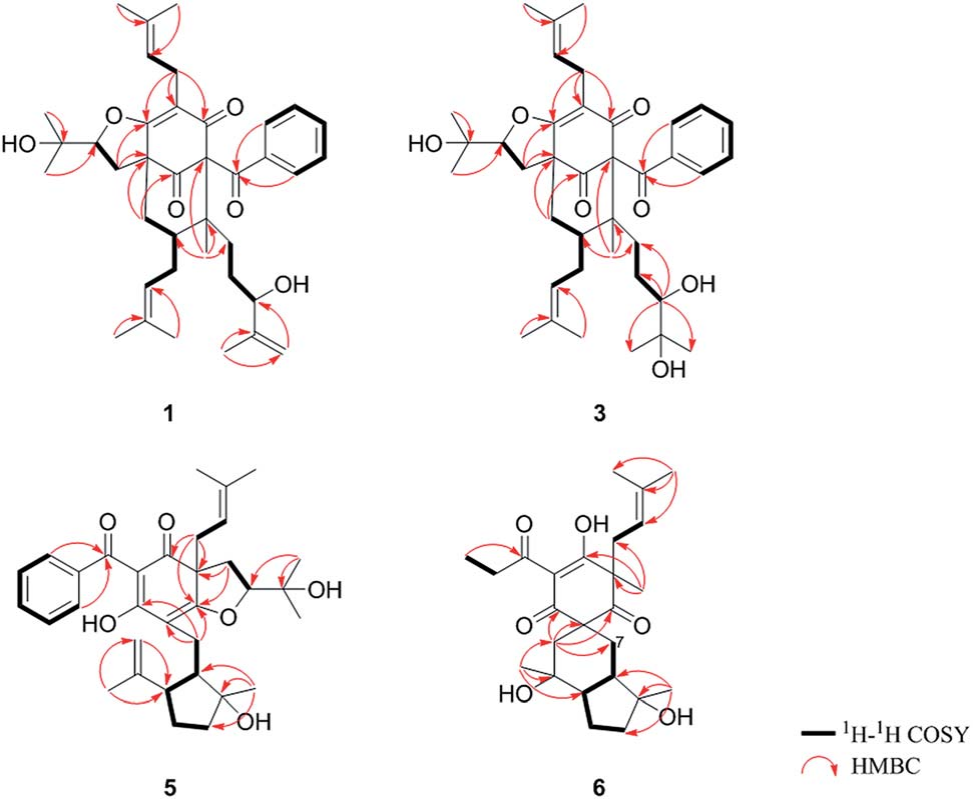
Key ^1^H–^1^H COSY and HMBC correlations of 1, 3, 5 and 6.

**Fig. 3:**
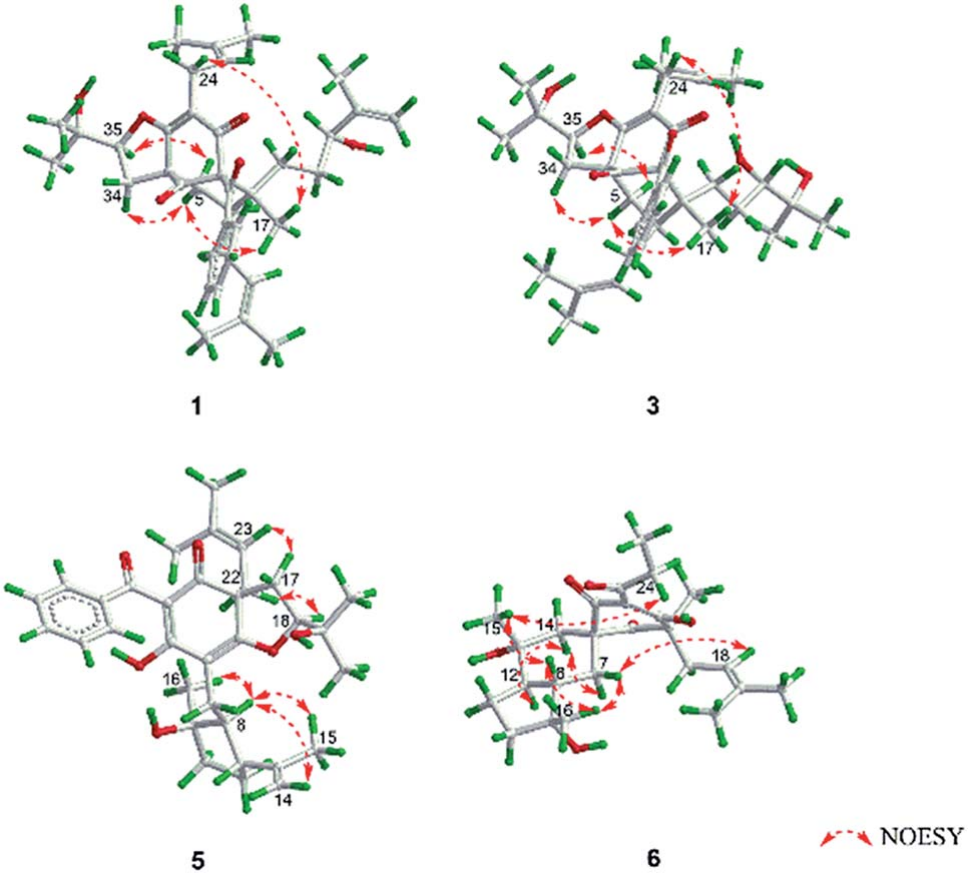
Key NOESY correlations of 1, 3, 5 and 6.

**Fig. 4:**
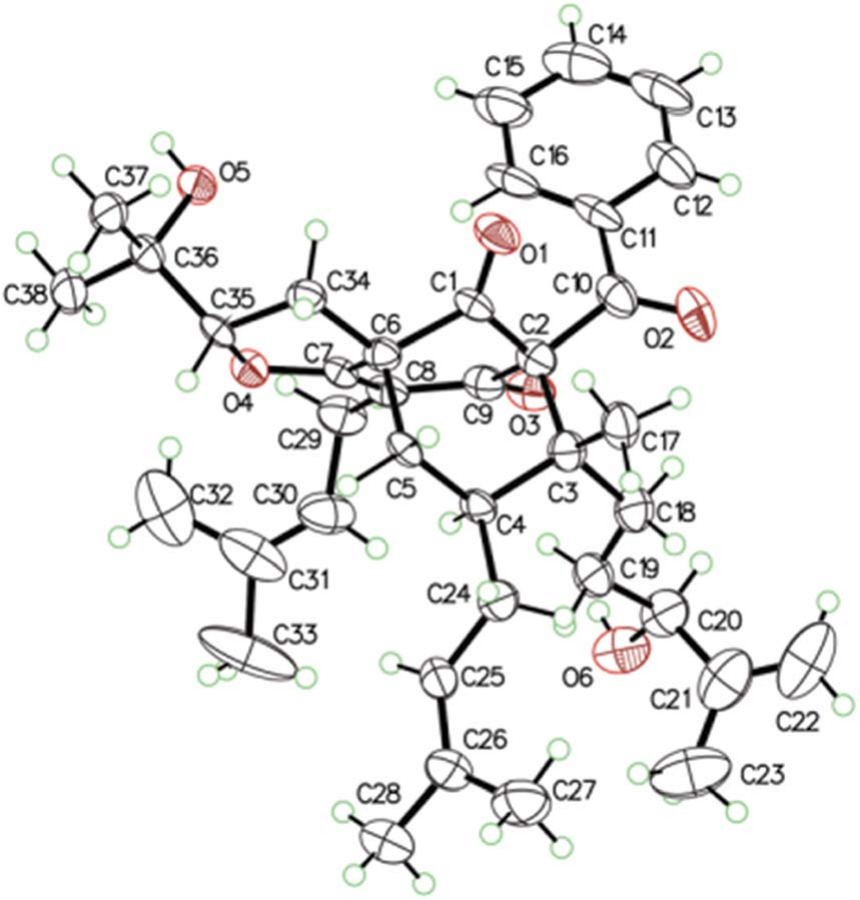
X-ray ORTEP drawing of 1.

Compound 1 was isolated from CH_3_OH as colorless crystals with [*α*]^25^_D_ +39.6 (*c* 1.0, MeOH). Its molecular formula was deduced as C_38_H_50_O_6_ on the basis of ^13^C NMR and HRESIMS (*m*/*z* 625.3515 [M + Na]^+^, calcd for C_38_H_50_NaO_6_ 625.3500) data. IR spectroscopy suggested the presence of hydroxyl (3456 cm^−1^), carbonyl (1716 cm^−1^) and aromatic double bond (1624, 1450 cm^−1^) groups. The NMR data of 1 (Table S1 and S2, ESI†) indicated the presence of an enolized 1,3-dicarbonyl ether group (*δ*_C_ 193.8, C-9; 116.3, C-8; 172.9, C-7), an unconjugated carbonyl carbon (*δ*_C_ 205.0, C-1), a methylene (*δ*_C_ 38.8, C-5), a methine (*δ*_C_ 43.2, C-4), and three quaternary carbons at *δ*_C_ 79.7 (C-2), 60.2 (C-6), and 49.7 (C-3), which suggested that 1 was a polycyclic polyprenylated acylphloroglucinol.^7,26,27^ Besides the above carbons, signals for eight methyls, six methylenes, nine methines and six quaternary carbons were observed. The NMR spectroscopic data of 1 resembled those of 32-*epi*-hyperforatin E.^27^ The main differences were that the absence of the 2-methylpropanoyl group [*δ*_H_ 2.00 (CH), 1.04 (CH_3_), 0.96 (CH_3_); *δ*_C_ 211.5 (C=O), 43.0 (CH), 21.8 (CH_3_), 20.8 (CH_3_)], and the presence of a benzoyl group [*δ*_H_ 7.41 (2CH), 7.36 (CH), 7.20 (2CH); *δ*_C_ 194.2 (C=O), 137.1 (C), 132.3 (CH), 128.3 (2CH), 128.1 (2CH)] in 1 (Fig. 1), which implied that the 2-methylpropanoyl group in 32-*epi*-hyperforatin E was replaced by a benzoyl group in 1. This was confirmed by the ^1^H–^1^H COSY cross-peaks between H-13/15 (*δ*_H_ 7.20) and H-12/16 (*δ*_H_ 7.41)/H-14 (*δ*_H_ 7.36), as well as the HMBC cross-peaks from H-12/16 to C-10 (*δ*_C_ 194.2)/C-14 (*δ*_C_ 132.3) (Fig. 2). The relative stereochemistry of 1 resembled those of 32-*epi*-hyperforatin E, basing on the NOESY correlations of Me-17 (*δ*_H_ 1.17) with H-5b (*δ*_H_ 1.63)/H-24, H-5b with H-34 and of H-5a (*δ*_H_ 2.10) with H-35 (*δ*_H_ 4.61) (Fig. 3). The absolute configuration at C-20 was confirmed by the induced ECD of the *in situ* formed [Rh_2_(OCOCF_3_)_4_] complex.^28,29^ According to the bulkiness rule,^28–30^ the 20*S* configuration of 1 was confirmed by the Cotton effect (positive E band) of the Rh complex (Fig. S1, ESI†). Additionally, the absolute configuration of 1 was unequivocally confirmed by X-ray crystallography (Fig. 4, CCDC 1865373) and ECD calculations (Fig. S2, ESI†), allowing the assignment of the absolute configuration of 1 as 2*R*, 3*R*, 4*S*, 6*S*, 20*S*, 35*S* (Fig. 1). Based on the above analysis, the structure of 1 was elucidated and named hyperpatulone A.

Compound 2 was isolated as a colorless oil with [*α*]^25^_D_ +41.7 (*c* 1.0, MeOH). The HRESIMS of compound 2 showed an [M + Na]^+^ ion peak at *m*/*z* 625.3506 (calcd for C_38_H_50_NaO_6_, 625.3500), consistent with the molecular formula of C_38_H_50_O_6_. Compounds 2 and 1 were separated by using chiral HPLC over a CHIRALPAK IC column. And the NMR spectroscopic data of 2 (Table S1 and S2, ESI†) was almost identical to those of 1, which indicated that 2 possessed the same planar structure as that of 1. However, compound 2 showed a negative E band in the *in situ* [Rh_2_(OCOCF_3_)_4_] complex-induced ECD spectrum (Fig. S1, ESI†), which is different from that of 1, suggesting an 20*R* configuration in compound 2. Thus, structure 2 was established, and named hyperpatulone B.

Compound 3 had the molecular formula C_38_H_52_O_7_, which was assigned by HRESIMS (*m*/*z* 643.3640 [M + Na]^+^, calcd for C_38_H_52_O_7_Na, 643.3605). According to its 1D NMR spectra (Tables S1 and S2, ESI†), compound 3 has the same skeleton as that of 1 except for the C-18-C-23 side chain. The differences between them were the absence of a terminal double bond (*δ*_C_ 147.9, 111.2) between C-21 and C-22, but the presence of one additional oxygenated quaternary carbon (*δ*_C_ 73.1) and one additional methyl group (*δ*_C_ 26.4) in 3, and the chemical shifts of C-18, 19, 20, 23 shifted from *δ*_C_ 32.7, 32.0, 76.5, 17.8 in 1 to *δ*_C_ 34.2, 28.2, 79.5, 23.8 in 3, which indicated the olefinic carbons (C-21, C-22) in 1 were replaced by a tertiary alcohol hydroxy group and a methyl group in 3. This was confirmed by the HMBC cross-peaks from H-20 (*δ*_H_ 3.26)/H-22 (*δ*_H_ 1.18)/H-23 (*δ*_H_ 1.12) to C-21 (*δ*_C_ 73.1) (Fig. 2). NOESY correlations of Me-17 (*δ*_H_ 1.18) with H-5b (*δ*_H_ 1.63)/H-24, of H-5b with H-34 and of H-5a (*δ*_H_ 2.10) with H-35 (*δ*_H_ 4.62) indicated that the relative configuration of 3 was identical to that of 1 (Fig. 3). The *in situ* [Rh_2_(OCOCF_3_)_4_] complex-induced ECD spectrum of 3 exhibited a positive E band for a 20*S* configuration (Fig. S3, ESI†). Therefore, structure 3 was determined and named hyperpatulone C.

The molecular formula of 4 was established to be C_33_H_42_O_6_ by its HRESIMS *m*/*z* 643.3626 [M + Na]^+^ (calcd for C_38_H_52_O_7_Na, 643.3605). The NMR data (Tables S1 and S2, ESI†) of 4 showed lots of similarities to those of 3, suggesting that 4 and 3 possessed the same planar structure. The only difference between 4 and 3 was the orientation of H-20, which was determined by a negative E band for a 20*R* configuration in the *in situ* [Rh_2_(OCOCF_3_)_4_] complex-induced ECD spectrum of 4 (Fig. S3, ESI†). Accordingly, compound 4 was elucidated and named hyperpatulone D.

The molecular formula C_33_H_42_O_6_ of compound 5 was assigned by HRESIMS (*m*/*z* 557.2893 [M + Na]^+^, calcd for C_33_H_42_O_6_Na, 557.2874). The 1D NMR data (Tables S1 and S3, ESI†) of 5 showed lots of similarities to those of hyperascyrone G,^18^ with a 6/6/5 tricyclic spiro ring system. The natural occurring polyprenylated spirocyclic acylphloroglucinol derivatives (PSAPs), with a 6/6/5 tricyclic spiro ring system, were a special subgroup of PPAPs. Detailed comparison of the NMR spectra of 5 with those of hyperascyrone G indicated the absence of a 3-methylbutanoyl group [*δ*_H_ 3.04 and 2.88 (CH_2_), 2.30 (CH), 1.01 (CH_3_), 0.98 (CH_3_); *δ*_C_ 197.6 (C=O), 45.9 (CH_2_), 27.1 (CH), 22.9 (CH_3_), 22.6 (CH_3_)] in hyperascyrone G, but the presence of a benzoyl group [*δ*_H_ 7.44 (2CH), 7.43 (CH), 7.37 (2CH); *δ*_C_ 191.5 (C=O), 136.6 (C), 131.3 (CH), 128.1 (2CH), 127.9 (2CH)] in 5. Thus, it could be deduced that the 3-methylbutanoyl group in hyperascyrone G was replaced by a benzoyl group in 5. This was confirmed by the ^1^H–^1^H COSY cross-peaks between H-30/32 (*δ*_H_ 7.37) and H-29/33 (*δ*_H_ 7.44)/H-31 (*δ*_H_ 7.43), as well as the HMBC cross-peaks from H-29/33 to C-27 (*δ*_C_ 191.5)/C-31 (*δ*_C_ 131.3) (Fig. 2). The relative configurations of 5 and hyperascyrone G were very similar by analysis of the NOESY correlations between H-18 (*δ*_H_ 4.55) and H-22a (*δ*_H_ 2.66), between H-23 (*δ*_H_ 5.13) and H-17a (*δ*_H_ 2.15), between H-8 (*δ*_H_ 1.83) and Me-15 (*δ*_H_ 1.71)/Me-16 (*δ*_H_ 1.18)/H-14a (*δ*_H_ 4.78) (Fig. 3). The ECD data obtained for 5 showed positive Cotton effects at *λ*_max_ 201 and 278 nm and a negative Cotton effect at *λ*_max_ 242 and 311 nm (Fig. S4, ESI†) comparable to those of hyperascyrone G.^18^ Thus, structure 5 was established, and named hyperpatulone E.

Compound 6 was assigned the molecular formula C_25_H_36_O_6_ by HRESIMS (*m*/*z* 455.2412 [M + Na]^+^, calcd for C_25_H_36_O_6_Na, 455.2404). The 1D NMR data (Tables S1 and S3, ESI†) of 6 showed lots of similarities to chipericumin D (14).^31^ Detailed comparison of the NMR spectra of 6 with those of chipericumin D indicated the absence of a 2-methylbutanoyl group [*δ*_H_ 3.16 (CH), 1.78 and 1.44 (CH_2_), 1.22 (CH_3_), 0.83 (CH_3_); *δ*_C_ 205.1 (C=O), 41.9 (CH), 25.3 (CH_2_), 19.5 (CH_3_), 12.3 (CH_3_)] in chipericumin D, but the presence of a propanoyl group [*δ*_H_ 2.98 (CH_2_), 0.97 (CH_3_); *δ*_C_ 201.0 (C=O), 46.2 (CH_2_), 23.0 (CH_3_)] in 6. Thus, it could be deduced that the 2-methylbutanoyl group in chipericumin D was replaced by a propanoyl group in 6. The structure was supported by the ^1^H–^1^H COSY correlations between H-24 (*δ*_H_ 2.98) and Me-25 (*δ*_H_ 0.97) together with the HMBC correlations between Me-25 and C-23 (*δ*_C_ 201.0) (Fig. 2). The relative configuration of 6 was same as that of chipericumin D with the analysis of the NOESY correlations of H-7a (*δ*_H_ 1.91)/H-14a (*δ*_H_ 1.45), H-12 (*δ*_H_ 1.78)/H-14a, H-8 (*δ*_H_ 1.67)/Me-15 (*δ*_H_ 0.95), H-7b (*δ*_H_ 1.76)/Me-16, H-8/Me-16 (*δ*_H_ 1.40), Me-15/H-24 (*δ*_H_ 2.98) and H-7b/H-18(*δ*_H_ 4.55) (Fig. 3). In addition, compounds 6 and chipericumin D (14) gave closely correlated Cotton effects in the ECD spectrum (Fig. S4, ESI†). Thus, structure 6 was established, and named hyperpatulone F.

Ten known compounds were identified as uralodin A (7),^32^ uralodin B (8),^13^ attenuatumione H (9),^26^ uralione D (10),^7^ uralione I (11),^7^ tomoeone A (12),^15^ tomoeone B (13),^15^ chipericumin D (14),^31^ hyperascyrone F (15),^18^ hypercohone G (16),^33^ by comparison of their spectroscopic and physical data with those of related literature.

The isolates 1–16 were tested for their cytotoxic activities by MTT assay on human HepG-2, HeLa, MCF-7 and A549 cell lines. Cisplatin was used as the positive control. As shown in Table 1, PSAPs compounds (5–6, 12–16) exhibited more potent cytotoxic activities than other PPAPs compounds (1–4, 7–11), with IC_50_ values of 9.52 ± 0.27 to 42.33 ± 1.91 μM. Especially, compound 5 shows significant cytotoxicities toward HepG-2, HeLa and A549 cell lines (IC_50_ = 9.52 ± 0.27, 11.87 ± 0.22 and 12.63 ± 0.12 μM).

**Table 1:** Cytotoxic activities of compounds 1–16

Compounds	IC_50_^a^ (μM)			A549
	HepG-2	HeLa	MCF-7	
1	>50	>50	>50	>50
2	>50	>50	46.83 ± 1.26	>50
3	>50	>50	>50	>50
4	>50	45.79 ± 1.21	>50	44.35 ± 0.62
5	9.52 ± 0.27	11.87 ± 0.22	20.83 ± 0.52	12.63 ± 0.12
6	26.73 ± 0.23	39.67 ± 0.27	42.33 ± 1.91	36.89 ± 0.81
7	>50	>50	>50	47.82 ± 1.17
8	41.03 ± 0.68	39.27 ± 1.23	35.72 ± 0.93	42.90 ± 1.04
9	>50	>50	>50	>50
10	>50	42.67 ± 0.42	39.31 ± 0.67	41.32 ± 1.32
11	>50	>50	42.97 ± 1.21	>50
12	30.91 ± 0.25	27.46 ± 0.37	35.29 ± 0.82	21.78 ± 0.57
13	35.67 ± 0.49	29.67 ± 0.21	31.44 ± 0.95	32.47 ± 0.31
14	22.83 ± 0.53	25.59 ± 0.32	26.92 ± 0.58	27.41 ± 0.71
15	29.38 ± 0.28	24.39 ± 0.28	27.37 ± 0.53	23.76 ± 0.17
16	19.28 ± 0.37	28.59 ± 0.35	22.91 ± 0.32	17.92 ± 0.23
Cisplatin^b^	5.9 ± 0.45	4.7 ± 0.17	6.7 ± 0.61	5.1 ± 0.21

a IC_50_ values of 1–16 were detected by MTT assay after incubation for 48 h; data are expressed as mean ± SD.

b Positive control.

## Experimental

### General experimental procedures

Optical rotations were obtained on a JASCO P-1020 polarimeter. UV spectra were recorded using a JASCO V-550 UV/VIS spectrophotometer. CD spectra were measured on a JASCO J-810 spectrometer. 1D and 2D NMR spectra were recorded on Bruker AV-500 NMR spectrometers with TMS as an internal standard. HRESIMS analyses were recorded on an Agilent 6210 ESI/TOF mass spectrometer. Column chromatography (CC) was performed with Silica gel (Qingdao Marine Chemical Plant, Qingdao, P. R. China), ODS (50 μm, YMC, Kyoto, Japan) and Sephadex LH-20 (Pharmacia Biotech, Uppsala, Sweden). Preparative HPLC was conducted on a Cosmosil C_18_ preparative column (5 μm, 20 × 250 mm) equipped with a G1311C pump and a G1315D photodiode array detector (Agilent Technologies, CA, USA). All chemical reagents were purchased from Tianjin Damao Chemical Company (Tianjin, P. R. China).

### Plant material

The whole plant of *Hypericum patulum* was collected in Guizhou Province of China, in August of 2016 and authenticated by Zhenqiu Mai, the senior engineer of Guangdong Province. A voucher specimen (no. 20160817) was deposited in the Institute of Traditional Chinese Medicine & Natural Products, Jinan University, Guangzhou, China.

### Extraction and isolation

The dried and powdered herbs of *Hypericum patulum* (12 kg) were extracted under reflux with 95% EtOH (30 L × 3) at room temperature. The combined ethanol extract was concentrated to afford a residue (654 g), which was suspended in water (4 L) and then extracted with petroleum ether (PE) (4 L × 3) and ethyl acetate (EtOAc) (4 L × 3). The PE extract (217 g) was subjected to silica gel column chromatography, eluting with PE-EtOAc (100 : 0 to 0 : 1, v/v) to yield seven fractions (Fr. A–F). Fr. C (18.7 g) was further applied to a silica gel CC with PE/EtOAc (10 : 1 to 1 : 1, v/v) to afford five subfractions (Fr. C1–C5). Fr. C2 (1.5 g) was purified by Sephadex LH-20 (CHCl_3_/MeOH, 2 : 1, v/v) and further separated by preparative HPLC (MeOH/H_2_O, 70 : 30, v/v) to yield compounds 1 (21.2 mg), 2 (18.5 mg), 7 (11.7 mg) and 8 (16.9 mg). Fr. C3 (7.5 g) was purified by ODS CC and Sephadex LH-20 to obtain compounds 3 (12.7 mg), 4 (13.9 mg), 9 (19.7 mg), 10 (15.7 mg) and 11 (13.2 mg). Fr. D (21.9 g) was applied to ODS CC using a MeOH/H_2_O gradient (40 : 60 to 100 : 0, v/v) to afford five subfractions (Fr. D.1–D.5). Fr. D.3 (3.6 g) was further purified by Sephadex LH-20 CC (CHCl_3_/MeOH, 1 : 1, v/v) and preparative HPLC (MeOH/H_2_O, 80 : 20, v/v) and to yield compounds 5 (9.5 mg), 12 (21.3 mg) and 13 (25.1 mg). Fr. D.4 (5.8 g) was separated by preparative HPLC (MeOH/H_2_O, 80 : 20, v/v) to yield compounds 6 (15.8 mg) and 14 (11.9 mg). Fr. D.5 (4.9 g) was purified by preparative HPLC (MeOH/H_2_O, 80 : 20, v/v) to achieve compounds 15 (9.2 mg) and 16 (15.2 mg).

#### Hyperpatulone A (1)

Colorless needle crystals (MeOH); mp 116–117 °C; [*α*]^25^_D_ +39.6 (*c* 1.0, MeOH); UV (MeOH) *λ*_max_ 203, 250 and 277 nm; IR (KBr) *ν*_max_ 3456, 2981, 2931, 1716, 1693, 1624, 1450, 1369, 1227 cm^−1^; ^1^H and ^13^C NMR spectroscopic data, see Tables S1 and S2 (ESI†); HRESIMS *m*/*z* 625.3515 [M + Na]^+^ (calcd for C_38_H_50_NaO_6_: 625.3500).

X-ray crystallographic analysis of 1 (Table S4, ESI†). C_38_H_50_O_6_, *M* = 602.78, orthorhombic, space group *P*2_1_2_1_2_1_; *a* = 19.2963(4) Å, *b* = 16.3762(4) Å, *c* = 11.0039(2) Å, *α* = 90°, *β* = 90°, *γ* = 90°, *V* = 3477.23(13) Å^3^, *T* = 100.00(10) K, *Z* = 4, *D*_calcd_ = 1.151 g m^−3^, *F* (000) = 1304.0. The final *R* values were *R*_1_ = 0.0809, *w*R_2_ = 0.2257, and the goodness of fit on *F*^2^ was equal to 1.156. Flack parameter = 0.0(2). The crystal data of compound 1 was deposited with the Cambridge Crystallographic Data Centre (CCDC 1865373, http://www.ccdc.cam.ac.uk/).†

#### Hyperpatulone B (2)

Colorless oil; [*α*]^25^_D_ +41.7 (*c* 1.0, MeOH); UV (MeOH) *λ*_max_ 204, 248 and 274 nm; IR (KBr) *ν*_max_ 3448, 2970, 2924, 1724, 1693, 1620, 1446, 1369, 1227 cm^−1^; ^1^H and ^13^C NMR spectroscopic data, see Tables S1 and S2 (ESI†); HRESIMS *m*/*z* 625.3506 [M + Na]^+^ (calcd for C_38_H_50_NaO_6_: 625.3500).

#### Hyperpatulone C (3)

Colorless oil; [*α*]^25^_D_ +56.6 (*c* 1.0, MeOH); UV (MeOH) *λ*_max_ 204, 247 and 274 nm; IR (KBr) *ν*_max_ 3425, 2977, 2931, 1705, 1600, 1442, 1389, 1273, 1215, 1119 cm^−1^; ^1^H and ^13^C NMR spectroscopic data, see Tables S1 and S2 (ESI†); HRESIMS *m*/*z* 643.3640 [M + Na]^+^ (calcd for C_38_H_52_O_7_Na: 643.3605).

#### Hyperpatulone D (4)

Colorless oil; [*α*]^25^_D_ +51.8 (*c* 1.0, MeOH); UV (MeOH) *λ*_max_ 203, 248 and 275 nm; IR (KBr) *ν*_max_ 3413, 2974, 2927, 1709, 1604, 1446, 1381, 1281, 1219, 1122 cm^−1^; ^1^H and ^13^C NMR spectroscopic data, see Tables S1 and S2 (ESI†); HRESIMS *m*/*z* 643.3626 [M + Na]^+^ (calcd for C_38_H_52_O_7_Na: 643.3605).

#### Hyperpatulone E (5)

Colorless oil; [*α*]^25^_D_ −37.5 (*c* 1.0, MeOH); UV (MeOH) *λ*_max_ 208, 242 and 353 nm; IR (KBr) *ν*_max_ 3413, 2965, 2927, 2877, 1716, 1612, 1454, 1376, 1269, 1153 cm^−1^; ^1^H and ^13^C NMR spectroscopic data, see Tables S1 and S3 (ESI†); HRESIMS *m*/*z* 557.2893 [M + Na]^+^ (calcd for C_33_H_42_O_6_Na: 557.2874).

#### Hyperpatulone F (6)

Colorless oil; [*α*]^25^_D_ +22.8 (*c* 1.0, MeOH); UV (MeOH) *λ*_max_ 203, 216, 249 and 279 nm; IR (KBr) *ν*_max_ 3410, 2970, 2935, 2877, 1720, 1662, 1612, 1454, 1385, 1319, 1273, 1211, 1153, 1107 cm^−1^; ^1^H and ^13^C NMR spectroscopic data, see Tables S1 and S3 (ESI†); HRESIMS *m*/*z* 455.2412 [M + Na]^+^ (calcd for C_25_H_36_O_6_Na, 455.2404).

### Cell culture

Human HepG-2, HeLa, MCF-7, and A549 cells were obtained from the Human Virology Institute of Sun Yat-Sen University. Cells were maintained in RPMI-1640 medium (Gibco, USA) supplemented with 10% fetal bovine serum (Gibco, USA) and 1% penicillin/streptomycin at 37 °C with 5% CO_2_ for 24 h.

### Cytotoxic assay *in vitro*

Four selected human cancer cell lines at the logarithmic phase were seeded in 96-well plates at 5 × 10^3^ cells per well, respectively. After incubating for 24 h, cells were treated with various concentrations of compounds 1–16 and incubated at 37 °C for 48 h. Then, the medium of each well was removed and 5 mg mL^−1^ MTT (30 μL) was added. After incubating for 4 h, the supernatant of each well was removed and DMSO (200 μL) was added to dissolve the formazan produced in the cells. The absorbance was recorded using an enzyme immunoassay reader (Thermo Labsystems Multiskan MK3) at 570 nm. The IC_50_ was calculated by the Bliss method: inhibitory rate = [(absorbance of the test group − absorbance of the blank control)/(absorbance of the control group-absorbance of the blank control)] × 100.

## Conclusions

In summary, six new PPAPs derivatives, hyperpatulone A–F (1–6), together with ten known analogs, were obtained from the dried herbs of *Hypericum patulum*. Their structures were determined by spectroscopic data, X-ray crystallography, ECD spectrum and Rh_2_(OCOCF_3_)_4_-induced ECD. Moreover, compounds 1–16 were evaluated for their cytotoxic activities on human HepG-2, HeLa, MCF-7, and A549 cell lines using the MTT assay. Compound 5 shows significant cytotoxicity toward HepG-2, HeLa and A549 cell lines (IC_50_ = 9.52 ± 0.27, 11.87 ± 0.22 and 12.63 ± 0.12 μM).

## Conflicts of interest

The authors declare no competing financial interest.

## Supplementary Material

RA-009-C9RA00277D-s001

RA-009-C9RA00277D-s002

## References

[cit1] Wu Q. L., Wang S. P., Wang L. W., Yang J. S., Xiao P. G. (1997). Nat. Prod. Res. Dev..

[cit2] Lv H. F., Chu Q. G., Hu H. Z. (2002). Chin. Tradit. Herb. Drugs.

[cit3] Xiao Z. Y., Mu Q. (2007). Nat. Prod. Res. Dev..

[cit4] Yin Z. Q., Wang Y., Zhang D. M., Ye W. C., Zhao S. X. (2004). Chinese Wild Plant Resources.

[cit5] Cui Y. H., Li J. (2006). J. Northeast Agric. Univ..

[cit6] Zhang L. S., Dong G. P., Liu G. M. (2009). J. Chin. Med. Mater..

[cit7] Zhou Z. B., Li Z. R., Wang X. B., Luo J. G., Kong L. Y. (2016). J. Nat. Prod..

[cit8] Ernst E., Rand J. I., Barnes J., Stevinson C. (1998). Eur. J. Clin. Pharmacol..

[cit9] Singer A., Wonnemann M., Müller W. E. (1999). J. Pharmacol. Exp. Ther..

[cit10] Butterweck V., Petereit F., Winterhoff H., Nahrstedt A. (1998). Planta Med..

[cit11] Chatterjee S. S., Bhattacharya S. K., Wonnemann M., Singer A., Muller W. E. (1998). Life Sci..

[cit12] Albert D., Zundorf I., Dingermann T., Muller W. E., Steinhilber D., Werz O. (2002). Biochem. Pharmacol..

[cit13] Chen X. Q., Li Y., Cheng X., Wang K., He J., Pan Z. H., Li M. M., Peng L. Y., Xu G., Zhao Q. S. (2010). Chem. Biodiversity.

[cit14] Hu L. H., Sim K. Y. (2000). Tetrahedron.

[cit15] Hashida W., Tanaka N., Kashiwada Y., Sekiya M., Ikeshiro Y., Takaishi Y. (2008). Phytochemistry.

[cit16] Zhang J. J., Yang X. W., Ma J. Z., Ye Y., Shen X. L., Xu G. (2015). Tetrahedron.

[cit17] Fobofou S. A. T., Franke K., Sanna G., Porzel A., Bullita E., Colla P. L., Wessjohann L. A. (2015). Bioorg. Med. Chem..

[cit18] Zhu H. C., Chen C. M., Liu J. J., Sun B., Wei G. Z., Li Y., Zhang J. W., Yao G. M., Luo Z. W., Xue Y. B., Zhang Y. H. (2015). Phytochemistry.

[cit19] Naonobu T., Yuki Y., Yutaka T., Yoshiki K. (2016). Org. Lett..

[cit20] Jayasariya H., Clark A. M., Mc C. J. D. (1991). J. Nat. Prod..

[cit21] Fobofou S. A. T., Harmon C. R., Lonfouo A. H. N., Franke K., Wright S. M., Wessjohann L. A. (2016). Phytochemistry.

[cit22] George A. K., Dan P., John T., Susan C. (1990). Biochem. Biophys. Res. Commun..

[cit23] Zhao J., Zhang Z. P., Chen H. S., Chen X. H. (1998). Acta Pharmacol. Sin..

[cit24] Gao W., Hou W. Z., Zhao J., Xu F., Li L., Xu F., Sun H., Xing J. G., Peng Y., Wang X. L., Ji T. F., Gu Z. Y. (2016). J. Nat. Prod..

[cit25] Yang X. W., Ding Y. Q., Zhang J. J., Liu X., Yang L. X., Li X. N., Ferreira D., Walker L. A., Xu G. (2014). Org. Lett..

[cit26] Zhou Z. B., Zhang Y. M., Luo J. G., Kong L. Y. (2016). Phytochem. Lett..

[cit27] Guo Y., Zhang N., Chen C. M., Huang J. F., Li X. N., Liu J. J., Zhu H. C., Tong Q. Y., Zhang J. W., Luo Z. W., Xue Y. B., Zhang Y. H. (2017). J. Nat. Prod..

[cit28] Frelek J., Szczepek W. J. (1999). Tetrahedron.

[cit29] Gerards M., Snatzke G. (1990). Tetrahedron.

[cit30] Liu L., Gao H., Chen X., Cai X., Yang L., Guo L., Yao X., Che Y. (2010). Eur. J. Org. Chem..

[cit31] Abe S., Tanaka N., Kobayashi J. (2012). J. Nat. Prod..

[cit32] Guo N., Chen X. Q., Zhao Q. S. (2008). Acta Bot. Yunnanica.

[cit33] Zhang J. J., Yang X. W., Ma J. Z., Liu X., Yang L. X., Yang S. C., Xu G. (2014). Nat. Prod. Bioprospect..

