# Supplementary material for: Hyperpatulones A–F, polycyclic polyprenylated acylphloroglucinols from Hypericum patulum and their cytotoxic activities
Source: RSC Adv. 2019 Mar 12;9(14):7961–6. doi: 10.1039/c9ra00277d (PMC9061578; doi:10.1039/c9ra00277d)
Supplement: RA-009-C9RA00277D-s001 [file RA-009-C9RA00277D-s001.pdf]

Electronic Supplementary Material (ESI) for RSC Advances.
This journal is © The Royal Society of Chemistry 2019

Supporting Information

Hyperpatulones A-F, polycyclic polyprenylated acylphloroglucinols from *Hypericum patulum* and their cytotoxic activities

Zhong-Nan Wu,[a] Qian-Wen Niu,[a] Yu-Bo Zhang,[ab] Ding Luo,[a] Qing-Guo Li,[c] Ying-Ying Li,[a] Guang-Kai Kuang,[a] Li-Jun He,[a] Guo-Cai Wang*[ab] and Yao-Lan Li*[a]

[a] Institute of Traditional Chinese Medicine & Natural Products, Guangdong Province Key Laboratory of Pharmacodynamic Constituents of TCM and New Drugs Research, College of Pharmacy, Jinan University, Guangzhou 510632, People's Republic of China

[b] Integrated Chinese and Western Medicine Postdoctoral Research Station, Jinan University, Guangzhou 510632, People's Republic of China

[c] School of Pharmaceutical Sciences, Guangzhou University of Chinese Medicine, Guangzhou 510006, China

List of Supporting information **Pages**

Table S1 ^{13}C NMR (125 MHz) spectroscopic data of **1-6** in $CDCl_3$ (δ in ppm)....1
Table S2 ^{1}H NMR (500 MHz) spectroscopic data of **1-4** in $CDCl_3$ (δ in ppm, J in Hz)....2
Table S3 ^{1}H NMR (500 MHz) spectroscopic data of **5-6** in $CDCl_3$ (δ in ppm, J in Hz)....3
Table S4 X-ray crystallographic data of **1**....4
Scheme S1 Differences between compounds **1**, **2** and 32-*epi*-Hyperforatin E....5
Scheme S2 Differences between compounds **3**, **4** and **1**....6
Scheme S3 Differences between compounds **5** and Hyperascyrone G....7
Scheme S4 Differences between compounds **6** and Chipericumin D....8
Figure S1 ECD spectra of the [$Rh_2(OCOCF_3)_4$] complexes of compounds **1** and **2** with the intrinsic ECD spectrum subtracted.....9
Figure S2 Calculated ECD spectrum of **1** and experimental ECD spectra of **1-4**....9
Figure S3 ECD spectra of the [$Rh_2(OCOCF_3)_4$] complexes of compounds **3** and **4** with the intrinsic ECD spectrum subtracted.....10
Figure S4 Experimental ECD spectra of **5**, **6** and **14**....10
Figure S5 HR-ESI-MS of **1**....11
Figure S6 UV spectrum of **1**....11
Figure S7 IR spectrum of **1**....12
Figure S8 ^{1}H NMR spectrum of **1**....12
Figure S9 ^{13}C NMR spectrum of **1**....13
Figure S10 DEPT-135 spectrum of **1**....13
Figure S11 ^{1}H-^{1}H COSY spectrum of **1**....14
Figure S12 HSQC spectrum of **1**....14
Figure S13 HMBC spectrum of **1**....15
Figure S14 NOESY spectrum of **1**....15
Figure S15 HR-ESI-MS of **2**....16
Figure S16 UV spectrum of **2**....16
Figure S17 IR spectrum of **2**....17
Figure S18 ^{1}H NMR spectrum of **2**....17
Figure S19 ^{13}C NMR spectrum of **2**....18
Figure S20 DEPT-135 spectrum of **2**....18
Figure S21 ^{1}H-^{1}H COSY spectrum of **2**....19
Figure S22 HSQC spectrum of **2**....19
Figure S23 HMBC spectrum of **2**....20
Figure S24 NOESY spectrum of **2**....20
Figure S25 HR-ESI-MS of **3**....21
Figure S26 UV spectrum of **3**....21
Figure S27 IR spectrum of **3**....22
Figure S28 ^{1}H NMR spectrum of **3**....22
Figure S29 ^{13}C NMR spectrum of **3**....23
Figure S30 DEPT-135 spectrum of **3**....23
Figure S31 ^{1}H-^{1}H COSY spectrum of **3**....24
Figure S32 HSQC spectrum of **3**....24
Figure S33 HMBC spectrum of **3**....25

Figure S34 NOESY spectrum of **3**......25
Figure S35 HR-ESI-MS of **4**......26
Figure S36 UV spectrum of **4**......26
Figure S37 IR spectrum of **4**......27
Figure S38 ^{1}H NMR spectrum of **4**......27
Figure S39 ^{13}C NMR spectrum of **4**......28
Figure S40 DEPT-135 spectrum of **4**......28
Figure S41 ^{1}H-^{1}H COSY spectrum of **4**......29
Figure S42 HSQC spectrum of **4**......29
Figure S43 HMBC spectrum of **4**......30
Figure S44 NOESY spectrum of **4**......30
Figure S45 HR-ESI-MS of **5**......31
Figure S46 UV spectrum of **5**......31
Figure S47 IR spectrum of **5**......32
Figure S48 ^{1}H NMR spectrum of **5**......32
Figure S49 ^{13}C NMR spectrum of **5**......33
Figure S50 DEPT-135 spectrum of **5**......33
Figure S51 ^{1}H-^{1}H COSY spectrum of **5**......34
Figure S52 HSQC spectrum of **5**......34
Figure S53 HMBC spectrum of **5**......35
Figure S54 NOESY spectrum of **5**......35
Figure S55 HR-ESI-MS of **6**......36
Figure S56 UV spectrum of **6**......36
Figure S57 IR spectrum of **6**......37
Figure S58 ^{1}H NMR spectrum of **6**......37
Figure S59 ^{13}C NMR spectrum of **6**......38
Figure S60 DEPT-135 spectrum of **6**......38
Figure S61 ^{1}H-^{1}H COSY spectrum of **6**......39
Figure S62 HSQC spectrum of **6**......39
Figure S63 HMBC spectrum of **6**......40
Figure S64 NOESY spectrum of **6**......40

Table S1 ^{13}C NMR (125 MHz) spectroscopic data of **1-6** in $CDCl_3$ (δ in ppm)

No.	**1**	**2**	**3**	**4**	**5**	**6**
1	205.0	205.0	204.9	204.8	195.2	198.8
2	79.7	79.5	79.4	79.4	107.0	113.5
3	49.7	50.0	50.3	50.0	193.1	196.9
4	43.2	42.6	42.4	42.3	106.9	66.5
5	38.8	38.9	38.8	38.7	176.7	207.7
6	60.2	60.2	60.2	60.3	60.8	56.5
7	172.9	172.7	173.0	173.4	20.8	23.4
8	116.3	116.3	116.3	116.4	50.8	48.1
9	193.8	193.7	193.9	194.1	80.1	79.5
10	194.2	194.2	194.5	194.3	41.0	39.8
11	137.1	137.1	137.0	137.0	28.2	21.8
12	128.3	128.4	128.4	128.3	52.7	52.3
13	128.1	128.1	128.1	128.1	147.6	73.6
14	132.3	132.3	132.4	132.4	110.8	51.3
15	128.1	128.1	128.1	128.1	19.3	20.8
16	128.3	128.4	128.4	128.4	28.3	27.0
17	14.6	15.1	15.3	15.2	29.8	39.8
18	32.7	32.6	34.2	33.4	91. 3	117.3
19	32.0	31.6	28.2	27.9	71.2	137.5
20	76.5	76.5	79.5	78.9	26.4	17.9
21	147.9	147.5	73.1	73.2	26.1	26.0
22	111.2	111.1	26.4	26.5	37.4	22.6
23	17.8	18.1	23.8	23.6	117.3	201.0
24	27.4	27.5	27.5	27.5	137.3	46.2
25	122.4	122.3	122.2	122.2	24.4	23.0
26	134.0	134.0	134.0	134.1	18.3	
27	26.2	26.2	26.2	26.2	191.5	
28	18.1	18.1	18.3	18.3	136.6	
29	22.4	22.4	22.3	22.3	128.1	
30	120.8	120.8	120.7	120.7	127.9	
31	132.8	132.8	132.9	132.9	131.3	
32	25.9	25.9	26.0	26.0	127.9	
33	18.3	18.3	18.1	18.1	128.1	
34	30.5	30.5	30.5	30.4		
35	90.4	90.4	90.5	90.6		
36	71.3	71.3	71.3	71.3		
37	26.8	26.8	26.8	26.9		
38	24.1	24.1	24.2	24.2		

Table S2 ^{1}H NMR (500 MHz) spectroscopic data of **1-4** in $CDCl_3$ (δ in ppm, J in Hz)

No.	**1**	**2**	**3**	**4**
1	-	-	-	-
2	-	-	-	-
3	-	-	-	-
4	1.82 m	1.83 m	1.82 m	1.82 m
5a	2.10 m	2.09 m	2.10 m	2.10 m
5b	1.63 overlapped	1.63 overlapped	1.63 overlapped	1.64 overlapped
6	-	-	-	-
7	-	-	-	-
8	-	-	-	-
9	-	-	-	-
10	-	-	-	-
11	-	-	-	-
12	7.41 d (7.2)	7.41 d (7.2)	7.41 d (7.4)	7.39 d (7.4)
13	7.20 t (7.6)	7.19 t (7.6)	7.20 t (7.6)	7.20 t (7.6)
14	7.36 t (7.2)	7.35 t (7.2)	7.36 t (7.4)	7.36 t (7.4)
15	7.20 t (7.6)	7.19 t (7.6)	7.20 t (7.6)	7.20 t (7.6)
16	7.41 d (7.2)	7.41 d (7.2)	7.41 d (7.4)	7.39 d (7.4)
17	1.17 s	1.16 s	1.18 s	1.18 s
18	2.09 m	2.09 m	2.09 m	2.07 m
	1.88 m	1.86 m	1.88 m	1.87 m
19	1.60 m	1.60 m	1.60 m	1.60 m
	1.49 m	1.49 m	1.45 m	1.46 m
20	3.98 t (6.1)	3.96 t (6.0)	3.26 d (9.0)	3.26 d (9.1)
21	-	-	-	-
22	4.94 br s	4.93 br s	1.17 s	1.18 s
	4.80 br s	4.80 br s		
23	1.71 s	1.70 s	1.13 s	1.12 s
24	2.11 m	2.12 m	2.14 m	2.17 m
	1.81 m	1.80 m	1.78 m	1.80 m
25	4.94 t (7.3)	4.96 t (7.3)	4.96 t (7.2)	4.95 t (7.2)
26	-	-	-	-
27	1.70 s	1.70 s	1.70 s	1.70 s
28	1.64 s	1.63 s	1.57 s	1.58 s
29	3.10 dd (14.5, 7.3)	3.12 dd (14.5, 7.3)	3.10 dd (15.3, 7.1)	3.10 dd (15.1, 7.0)
	3.05 dd (14.5, 7.3)	3.03 dd (14.5, 7.3)	3.05 dd (15.3, 7.1)	3.06 dd (15.1, 7.0)
30	5.05 t (7.3)	5.05 t (7.3)	5.04 t (7.1)	5.03 t (7.0)
31	-	-	-	-
32	1.65 s	1.65 s	1.65 s	1.65 s
33	1.71 s	1.74 s	1.64 s	1.64 s
34	2.66 dd (13.2, 10.3)	2.67 dd (13.3, 10.7)	2.68 m	2.67 m
	1.83 dd (13.2, 6.0)	1.83 dd (13.3, 6.0)	1.83 m	1.82 m
35	4.61 dd (10.3, 6.0)	4.61 dd (10.7, 6.0)	4.62 dd (10.3, 5.9)	4.62 dd (10.3, 5.9)
36	-	-	-	-
37	1.37 s	1.37 s	1.38 s	1.38 s
38	1.21 s	1.21 s	1.21 s	1.21 s

Table S3 ^{1}H NMR (500 MHz) spectroscopic data of **5-6** in $CDCl_3$ (δ in ppm, J in Hz)

Position	**5**	**6**
1	-	-
2	-	-
3	-	-
4	-	-
5	-	-
6	-	-
7a	2.47 m	1.91 t (12.5)
7b	2.03 m	1.76 m
8	1.83 m	1.67 m
9	-	-
10	1.76 m	1.75 m
	1.72 m	
11a	1.44 m	1.87 m
11b	1.93 m	1.39 m
12	2.57 m	1.78 m
13	-	-
14a	4.78 (d, 3.0)	1.45 d (13.4)
14b	4.73 (d, 3.0)	2.32 d (13.4)
15	1.71 s	0.95 s
16	1.18 s	1.40 s
17a	2.15 m	2.67 dd (13.9, 7.7)
17b	1.25 m	2.35 dd (13.9, 7.7)
18	4.55 dd (10.5, 5.0)	4.55 brt (7.7)
19	-	-
20	1.28 s	1.39 s
21	1.16 s	1.49 s
22a	2.66 m	1.48 s
22b	2.47 m	
23	5.13 m	-
24	-	2.98 m
25	1.76 s	0.97 m
26	1.63 s	
27	-	
28	-	
29	7.44 m	
30	7.37 m	
31	7.43 m	
32	7.37 m	
33	7.44 m	

Table S4 X-ray crystallographic data of **1**

Empirical formula	$C_{38}H_{50}O_6$
Formula weight	602.78
Temperature/K	100.00(10)
Crystal system	orthorhombic
Space group	$P2_12_12_1$
a/Å	19.2963(4)
b/Å	16.3762(4)
c/Å	11.0039(2)
α/°	90
β/°	90
γ/°	90
Volume/Å^3	3477.23(13)
Z	4
ρ_{calc}g/cm^3	1.151
μ/mm^{-1}	0.607
F(000)	1304
Radiation	CuKα (λ = 1.54184)
2Θ range for data collection/°	7.080 to 147.676
Index ranges	$-23 \leq h \leq 23$, $-17 \leq k \leq 19$, $-13 \leq l \leq 9$
Reflections collected	18143
Independent reflections	6796 [R_{int} = 0.0972, R_{sigma} = 0.0809]
Data/restraints/parameters	6796/0/407
Goodness-of-fit on F^2	1.147
Final R indexes [I>=2σ (I)]	R_1 = 0.0809, wR_2 = 0.2226
Final R indexes [all data]	R_1 = 0.0972, wR_2 = 0.2310
Largest diff. peak/hole / e Å^{-3}	0.451/-0.344
Flack parameter	0.0(2)

Scheme S1 Differences between compounds **1**, **2** and 32-*epi*-Hyperforatin E

No.	**1**	**2**	32-*epi*-Hyperforatin E
1	205.0	205.0	205.8
2	79.7	79.5	84.5
3	49.7	50.0	49.2
4	43.2	42.6	44.4
5	38.8	38.9	39.1
6	60.2	60.2	61.0
7	172.9	172.7	176.0
8	116.3	116.3	117.6
9	193.8	193.7	194.7
10	194.2	194.2	211.5
11	137.1	137.1	43.0
12	128.3	128.4	21.8
13	128.1	128.1	20.8
14	132.3	132.3	
15	128.1	128.1	
16	128.3	128.4	
17	14.6	15.1	14.6
18	32.7	32.6	34.1
19	32.0	31.6	32.5
20	76.5	76.5	77.8
21	147.9	147.5	148.8
22	111.2	111.1	111.5
23	17.8	18.1	17.6
24	27.4	27.5	28.0
25	122.4	122.3	123.5
26	134.0	134.0	134.5
27	26.2	26.2	25.9
28	18.1	18.1	18.1
29	22.4	22.4	23.0
30	120.8	120.8	122.6
31	132.8	132.8	133.2
32	25.9	25.9	26.1
33	18.3	18.3	18.1
34	30.5	30.5	30.8
35	90.4	90.4	92.1
36	71.3	71.3	71.3
37	26.8	26.8	26.2
38	24.1	24.1	25.5

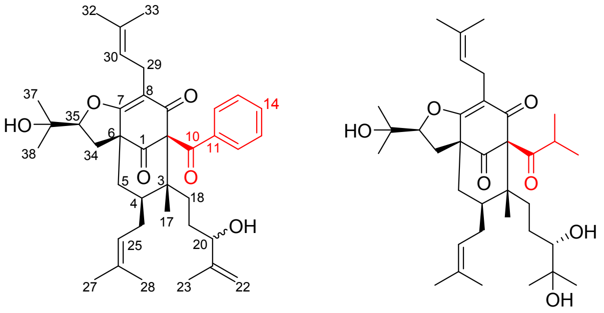

1 20*S*
2 20*R*

32-*epi*-Hyperforatin E

Scheme S2 Differences between compounds **3**, **4** and **1**

No.	**3**	**4**	**1**
1	204.9	204.8	205.0
2	79.4	79.4	79.7
3	50.3	50.0	49.7
4	42.4	42.3	43.2
5	38.8	38.7	38.8
6	60.2	60.3	60.2
7	173.0	173.4	172.9
8	116.3	116.4	116.3
9	193.9	194.1	193.8
10	194.5	194.3	194.2
11	137.0	137.0	137.1
12	128.4	128.3	128.3
13	128.1	128.1	128.1
14	132.4	132.4	132.3
15	128.1	128.1	128.1
16	128.4	128.4	128.3
17	15.3	15.2	14.6
18	34.2	33.4	32.7
19	28.2	27.9	32.0
20	79.5	78.9	76.5
21	73.1	73.2	147.9
22	26.4	26.5	111.2
23	23.8	23.6	17.8
24	27.5	27.5	27.4
25	122.2	122.2	122.4
26	134.0	134.1	134.0
27	26.2	26.2	26.2
28	18.3	18.3	18.1
29	22.3	22.3	22.4
30	120.7	120.7	120.8
31	132.9	132.9	132.8
32	26.0	26.0	25.9
33	18.1	18.1	18.3
34	30.5	30.4	30.5
35	90.5	90.6	90.4
36	71.3	71.3	71.3
37	26.8	26.9	26.8
38	24.2	24.2	24.1

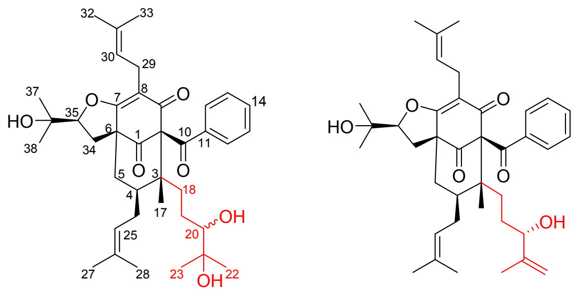

3 20*S*
4 20*R*

1

Scheme S3 Differences between compounds **5** and Hyperascyrone G

No.	**5**	Hyperascyrone G
1	195.2	196.1
2	107.0	108.6
3	193.1	194.1
4	106.9	106.6
5	176.7	176.3
6	60.8	61.3
7	20.8	22.2
8	50.8	49.9
9	80.1	79.6
10	41.0	42.1
11	28.2	28.6
12	52.7	53.6
13	147.6	148.0
14	110.8	111.0
15	19.3	19.1
16	28.3	28.4
17	29.8	30.5
18	91. 3	91. 3
19	71.2	70.0
20	26.4	27.4
21	26.1	25.5
22	37.4	38.5
23	117.3	118.4
24	137.3	136.4
25	24.4	25.9
26	18.3	17.8
27	191.5	197.9
28	136.6	45.9
29	128.1	27.0
30	127.9	22.7
31	131.3	22.9
32	127.9	
33	128.1	

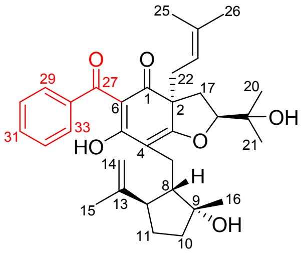

5

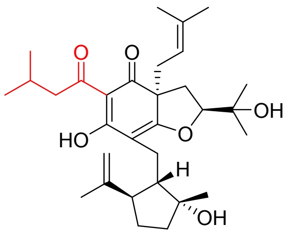

Hyperascyrone G

Scheme S4 Differences between compounds **6** and Chipericumin D

No.	**6**	Chipericumin D
1	198.8	199.4
2	113.5	112.4
3	196.9	196.4
4	66.5	66.5
5	207.7	207.9
6	56.5	56.4
7	23.4	23.2
8	48.1	47.8
9	79.5	79.3
10	39.8	39.5
11	21.8	21.6
12	52.3	52.0
13	73.6	73.4
14	51.3	51.1
15	20.8	20.3
16	27.0	26.7
17	39.8	40.4
18	117.3	117.1
19	137.5	137.3
20	17.9	17.5
21	26.0	25.8
22	22.6	22.4
23	201.0	205.1
24	46.2	41.9
25	23.0	19.5
26		25.3
27		12.3

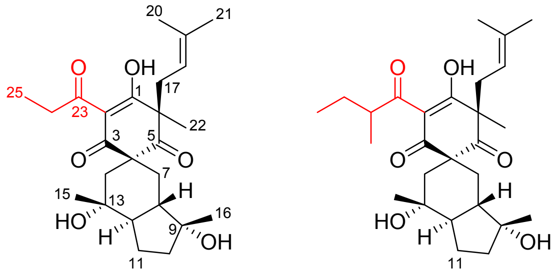

6

Chipericumin D

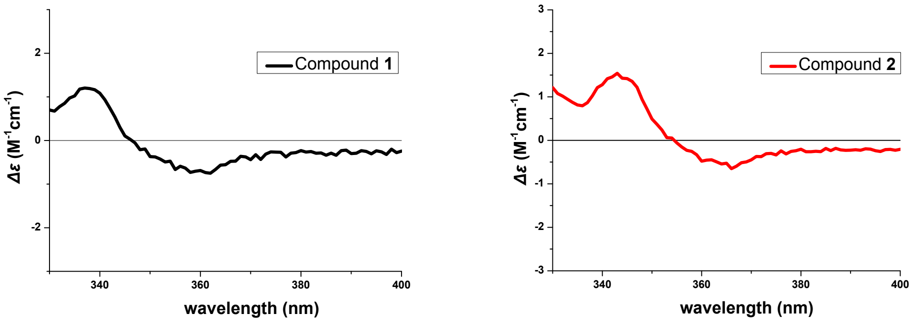

Figure S1 ECD spectra of the $[Rh_2(OCOCF_3)_4]$ complexes of compounds **1** and **2** with the intrinsic ECD spectrum subtracted.

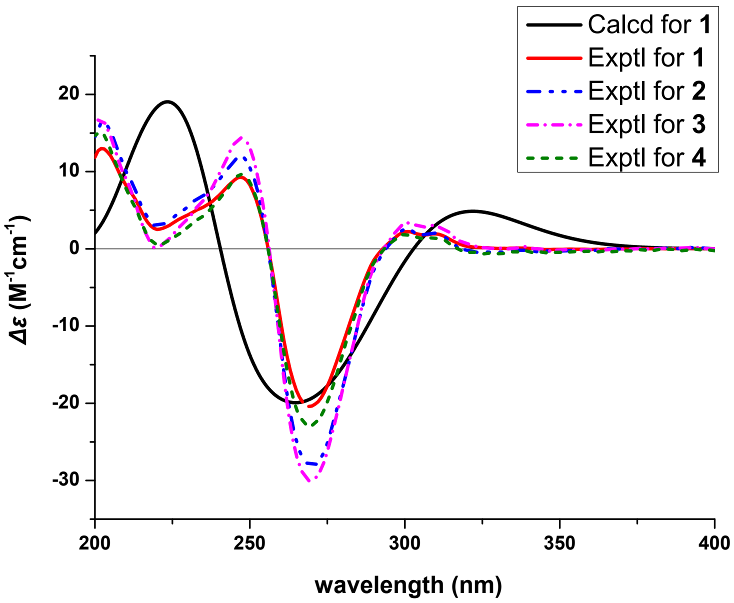

Figure S2 Calculated ECD spectrum of **1** and experimental ECD spectra of **1-4**

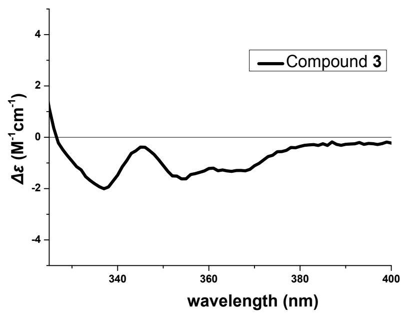

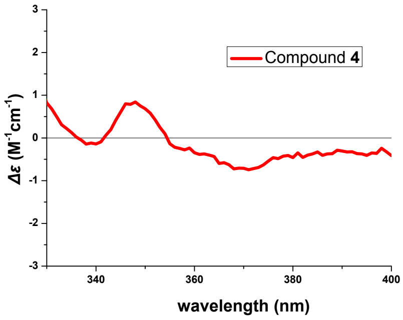

Figure S3 ECD spectra of the $[Rh_2(OCOCF_3)_4]$ complexes of compounds **3** and **4** with the intrinsic ECD spectrum subtracted.

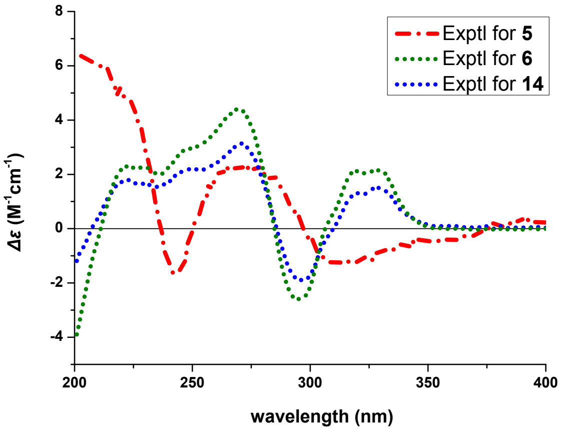

Figure S4 Experimental ECD spectra of **5**, **6** and **14**

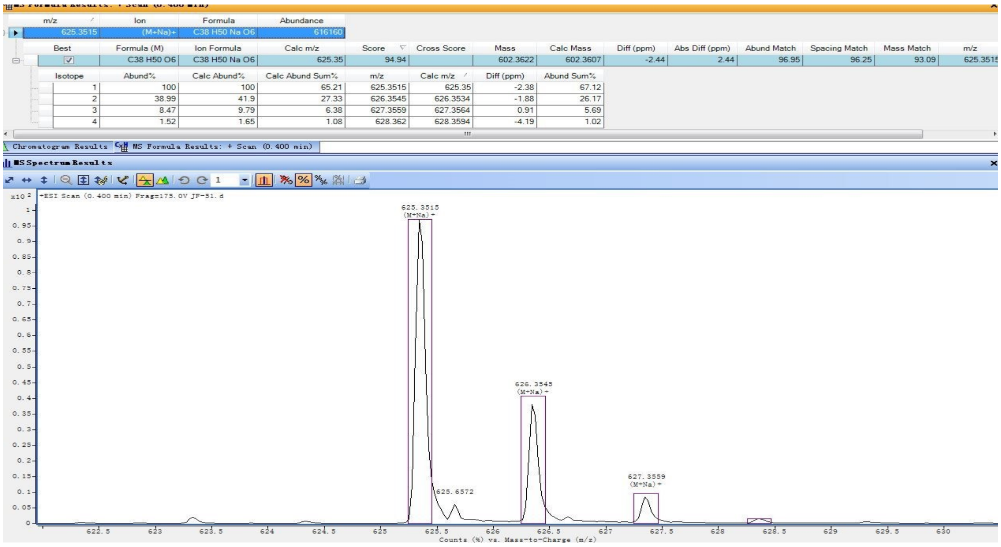

Figure S5 HR-ESI-MS of **1**

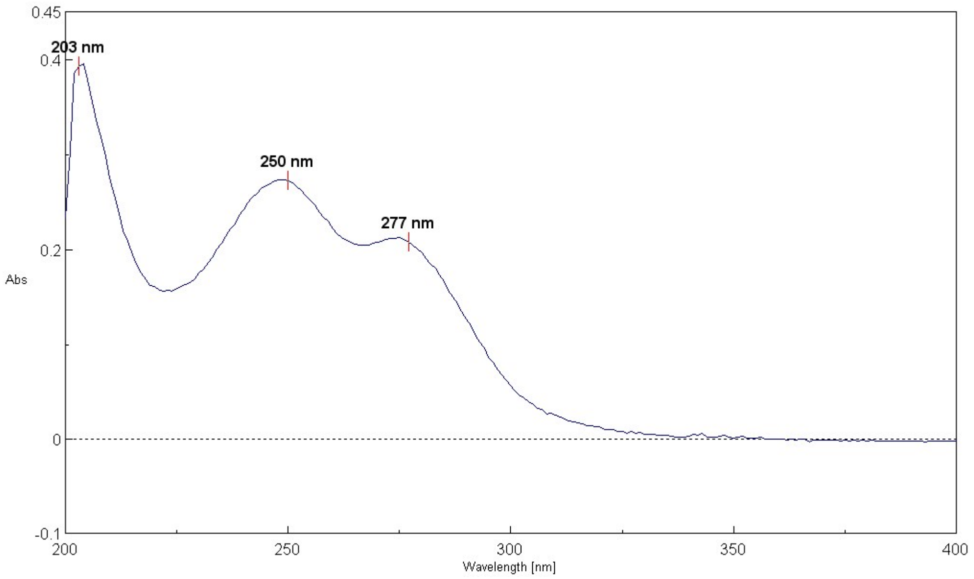

Figure S6 UV spectrum of **1**

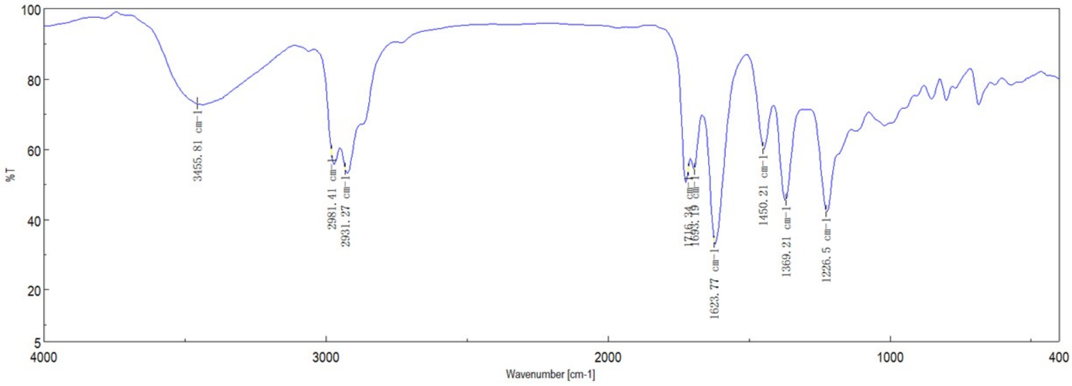

Figure S7 IR spectrum of **1**

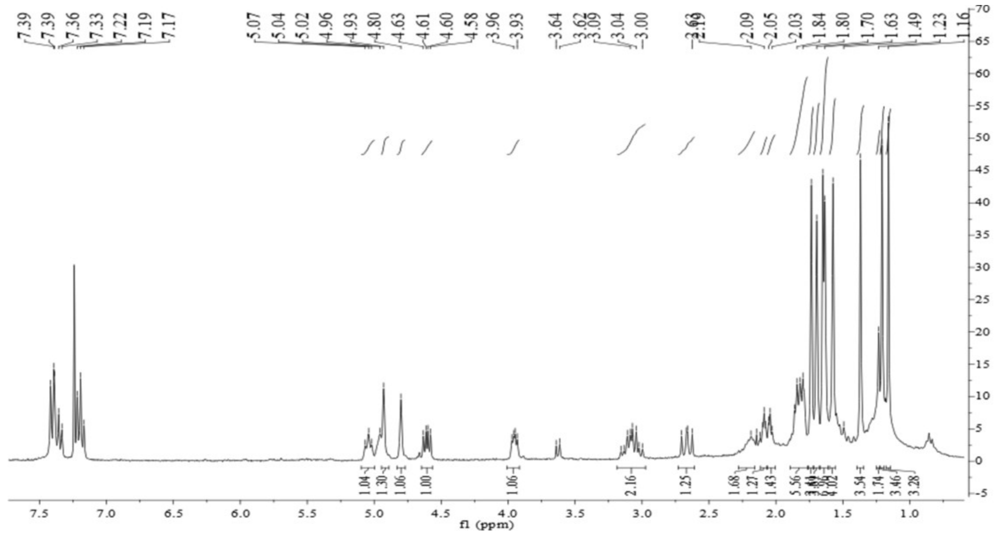

Figure S8 ^{1}H NMR spectrum of **1**

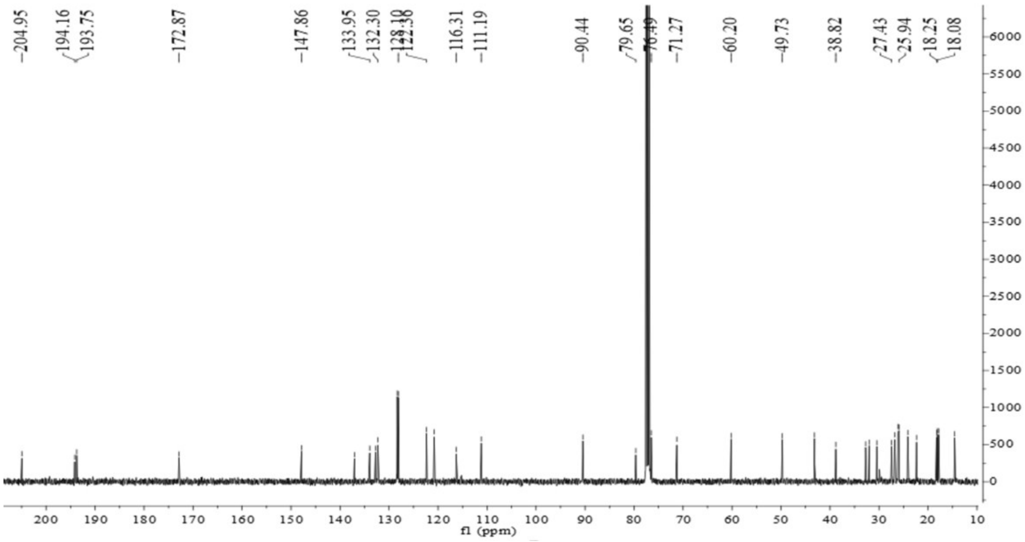

Figure S9 ^{13}C NMR spectrum of **1**

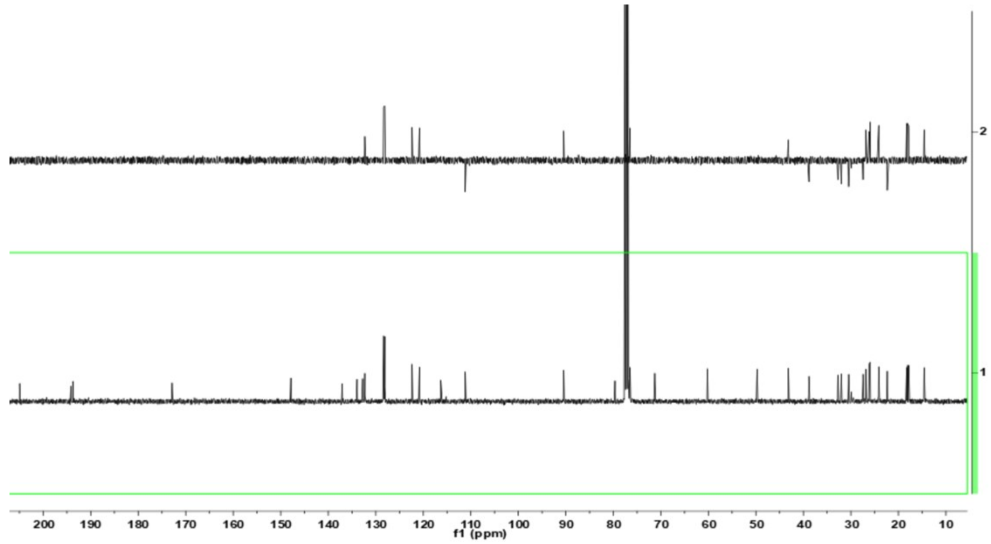

Figure S10 DEPT-135 spectrum of **1**

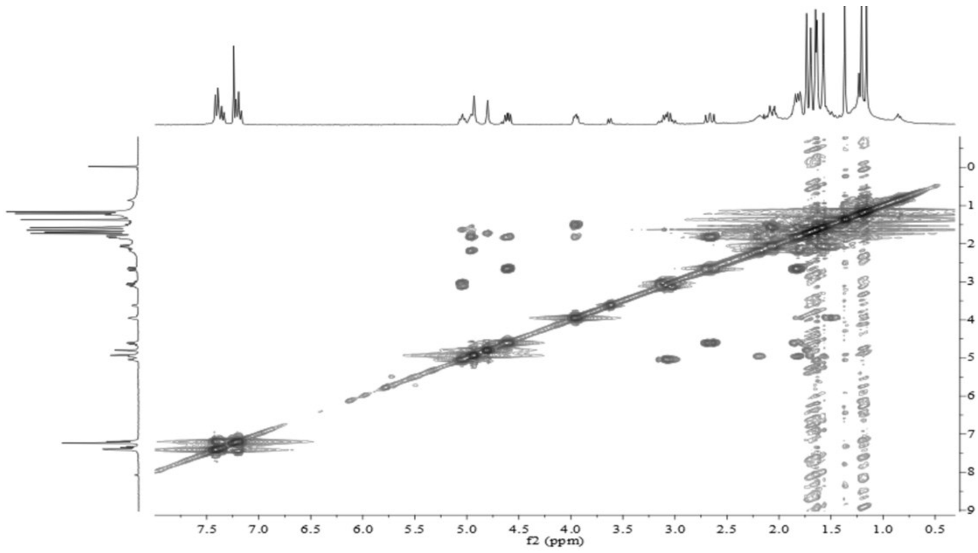

Figure S11 ^{1}H-^{1}H COSY spectrum of **1**

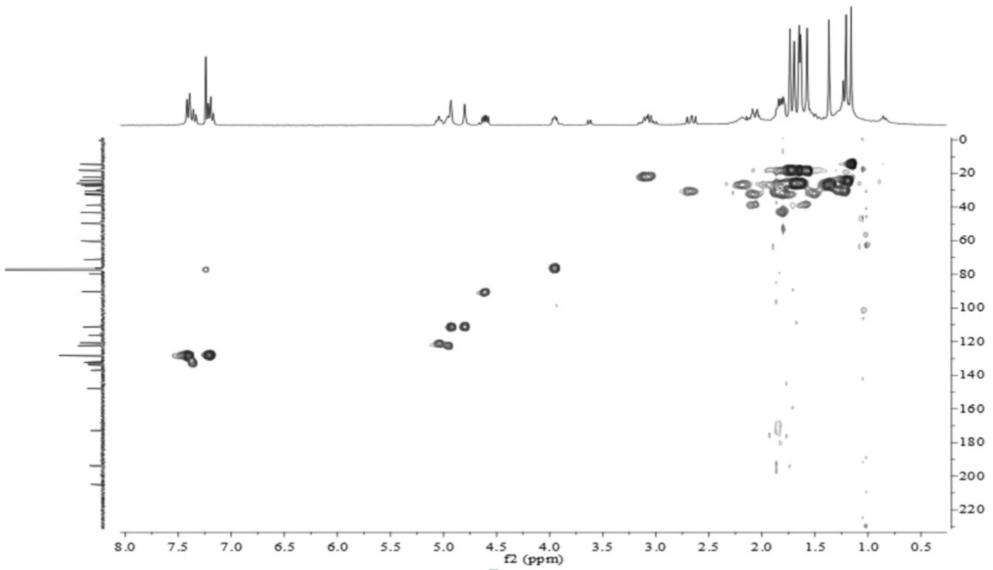

Figure S12 HSQC spectrum of **1**

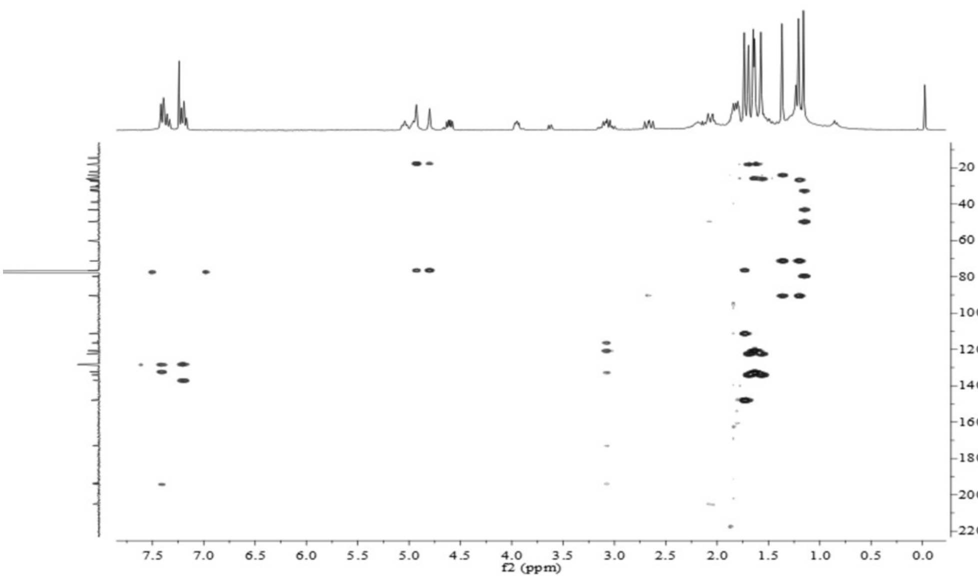

Figure S13 HMBC spectrum of **1**

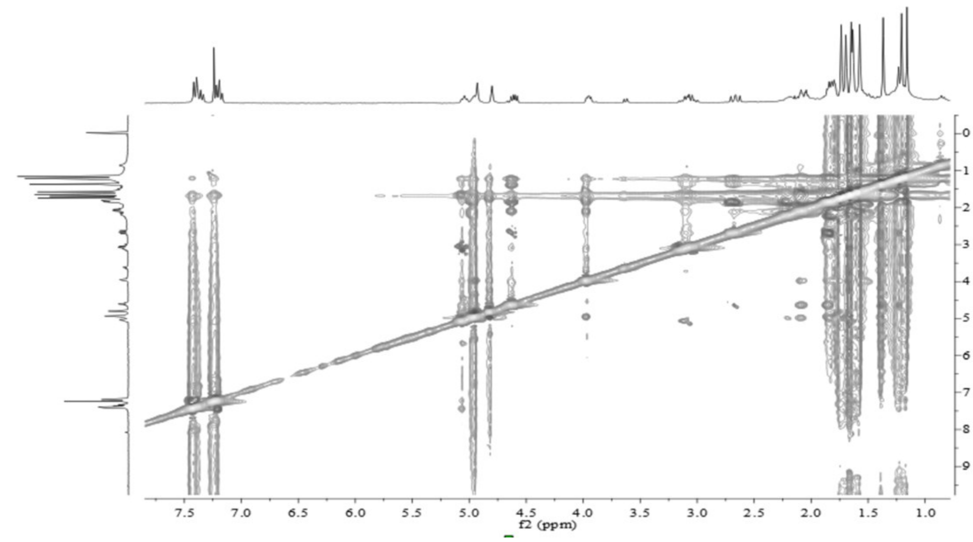

Figure S14 NOESY spectrum of **1**

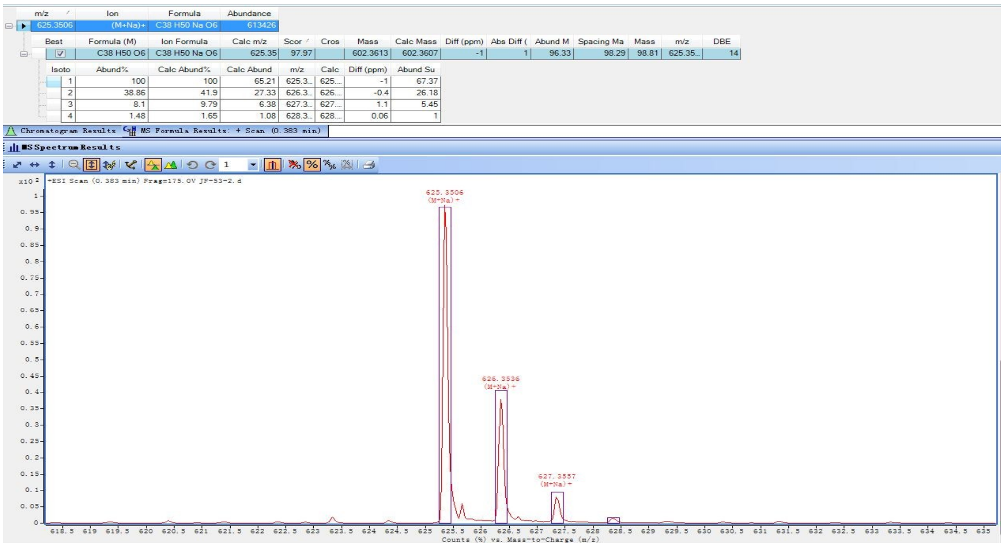

Figure S15 HR-ESI-MS of **2**

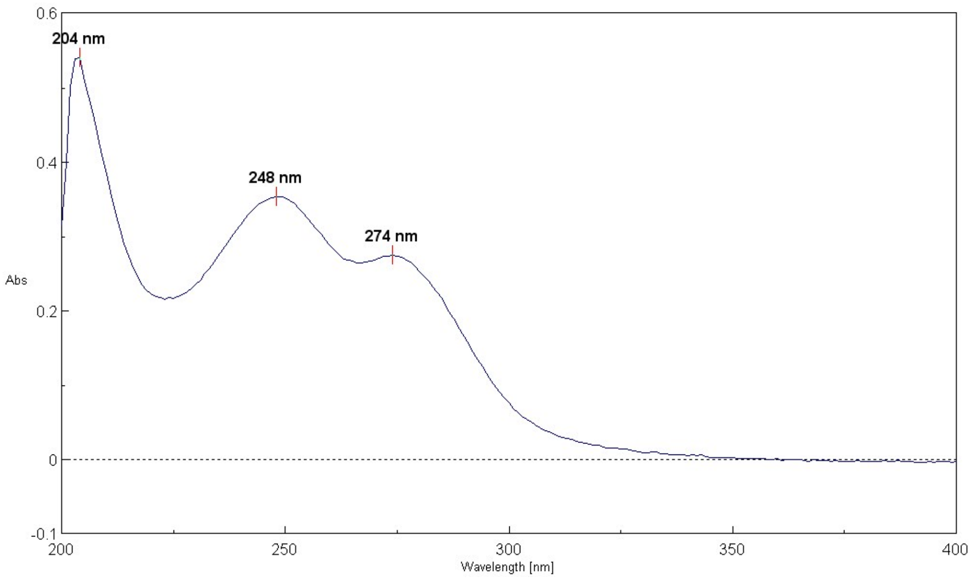

Figure S16 UV spectrum of **2**

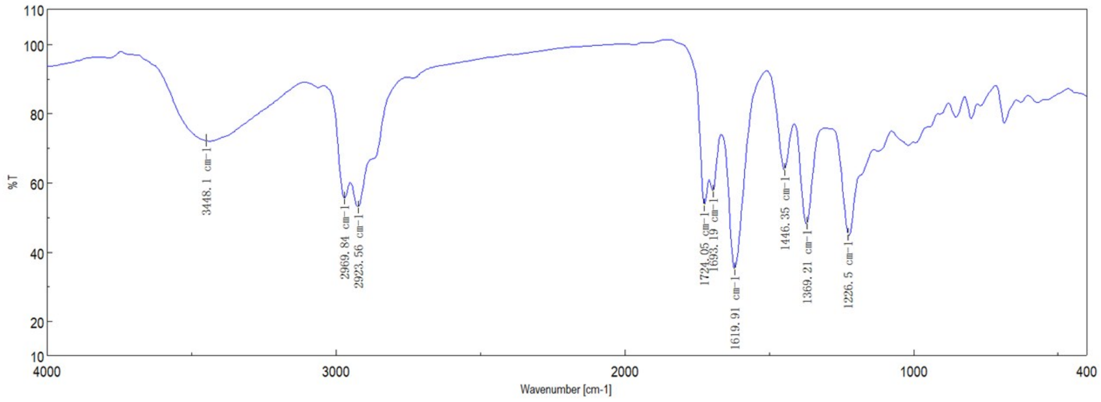

Figure S17 IR spectrum of **2**

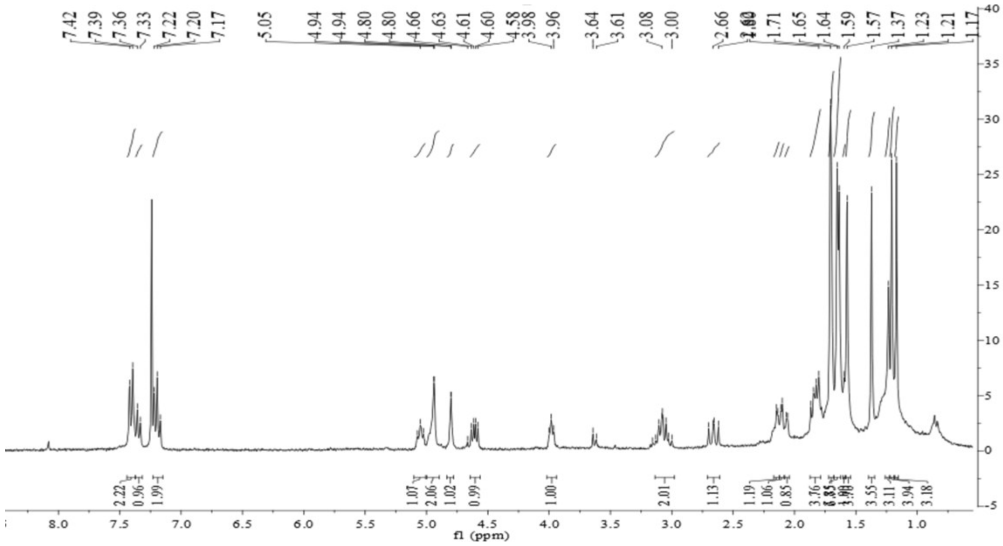

Figure S18 ^{1}H NMR spectrum of **2**

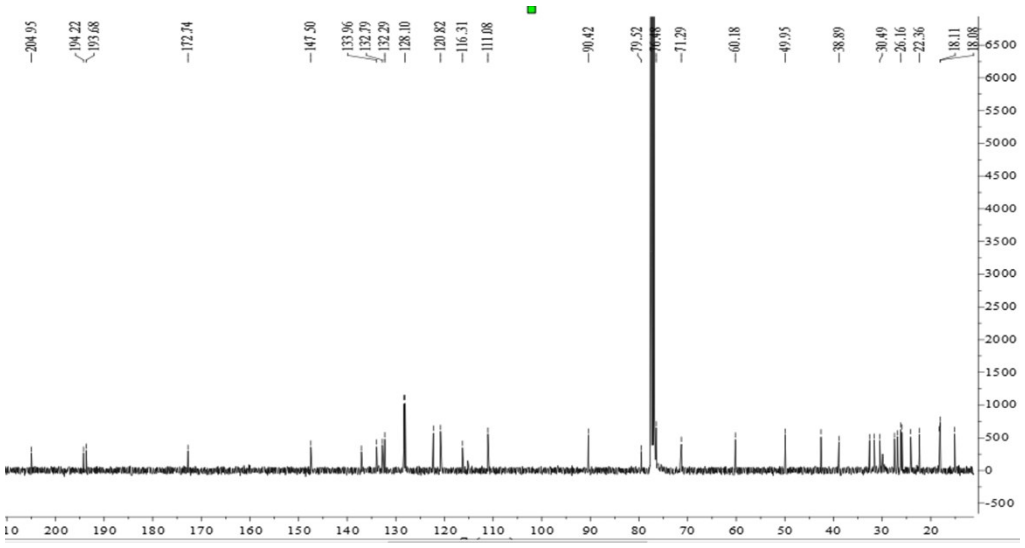

Figure S19 ^{13}C NMR spectrum of **2**

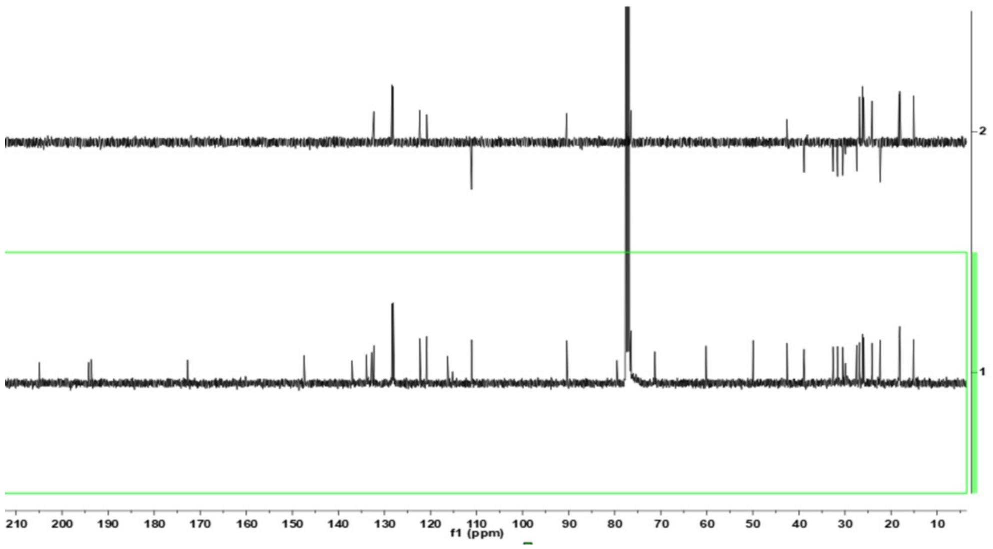

Figure S20 DEPT-135 spectrum of **2**

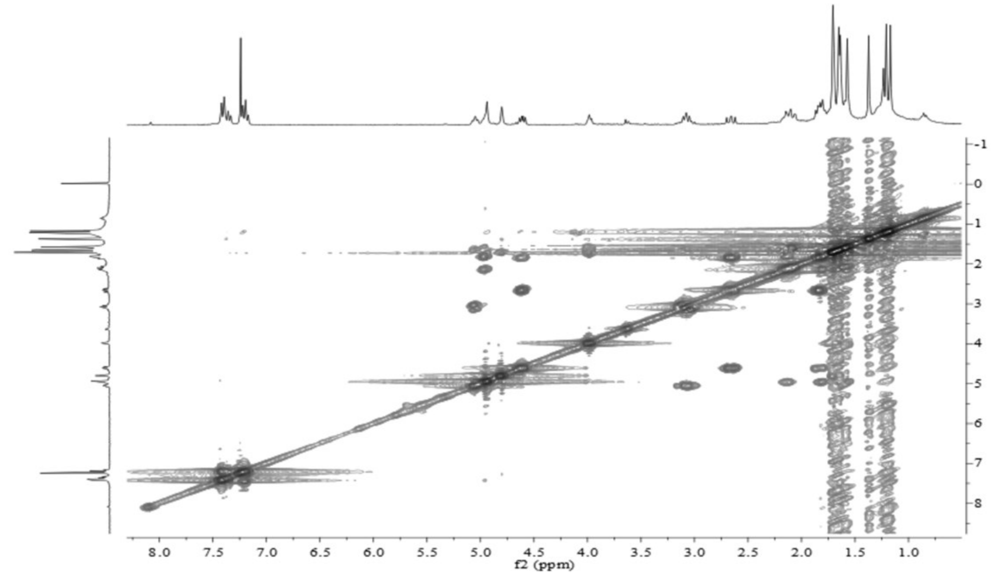

Figure S21 ^{1}H-^{1}H COSY spectrum of **2**

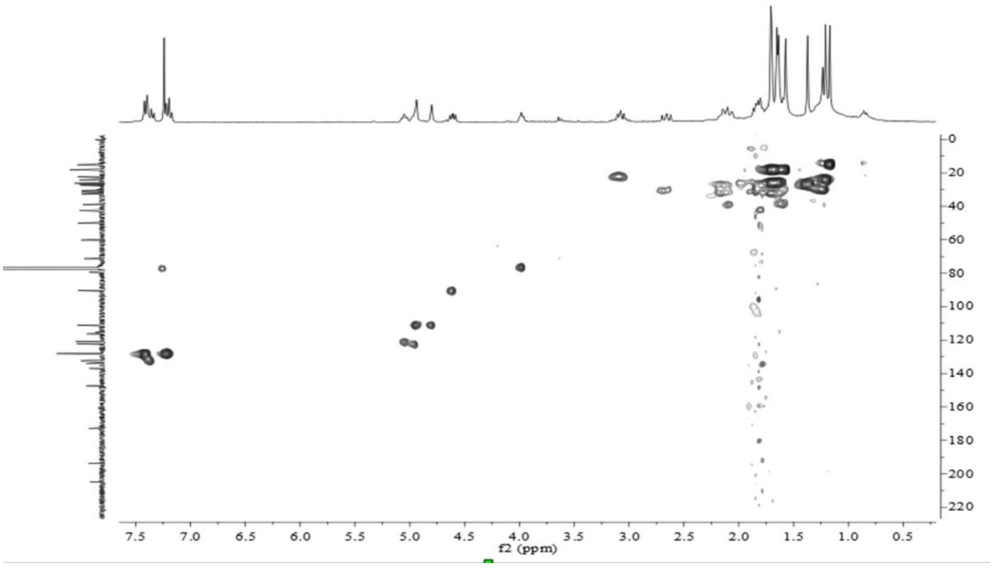

Figure S22 HSQC spectrum of **2**

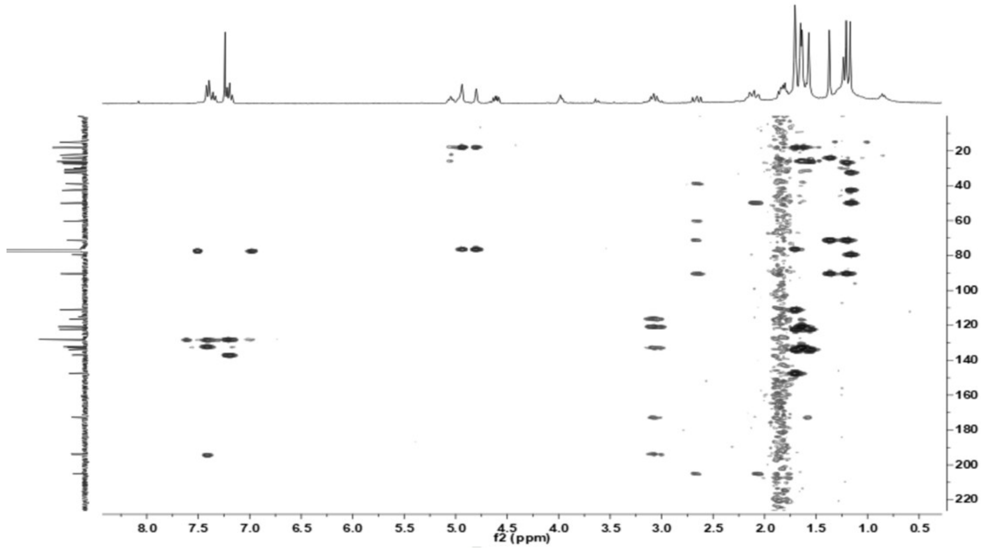

Figure S23 HMBC spectrum of **2**

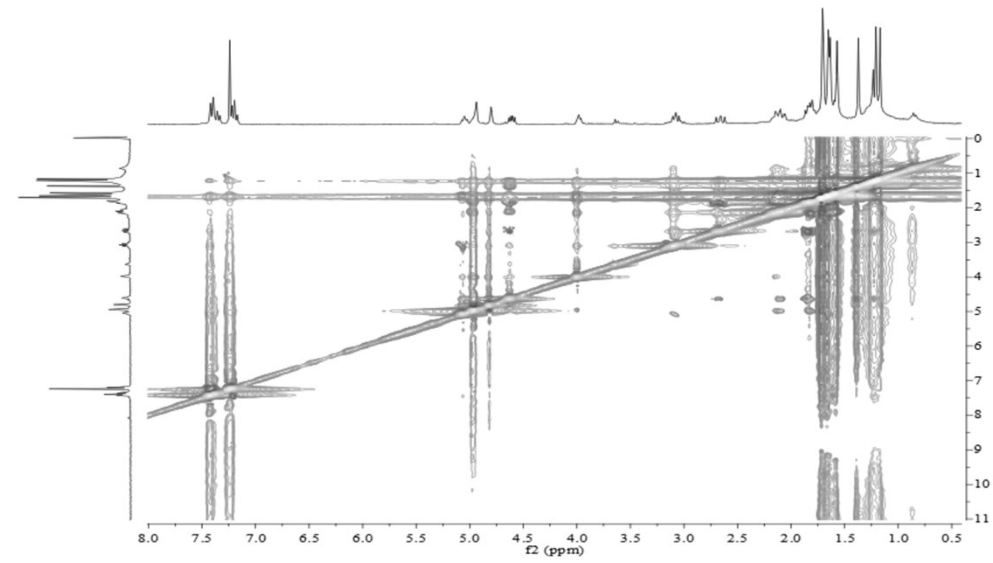

Figure S24 NOESY spectrum of **2**

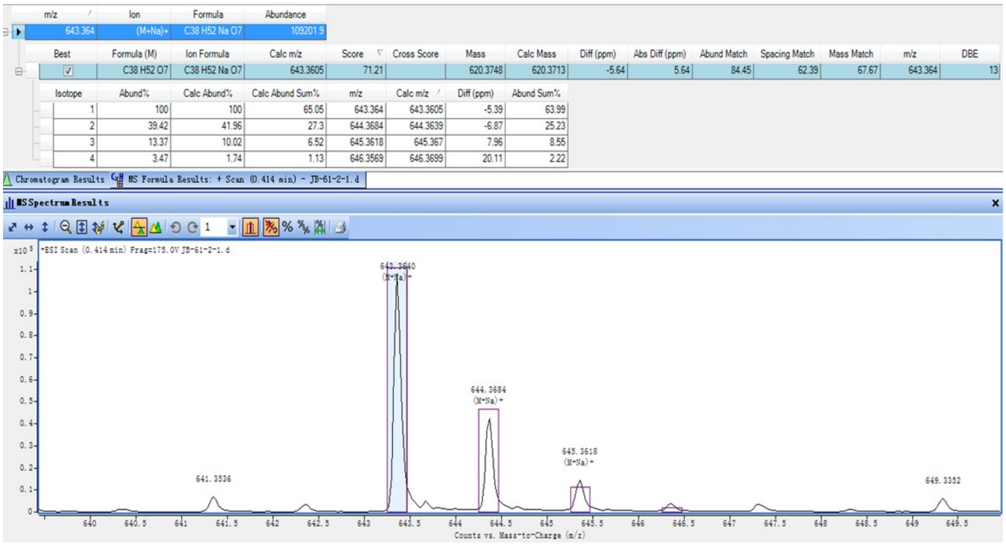

Figure S25 HR-ESI-MS of **3**

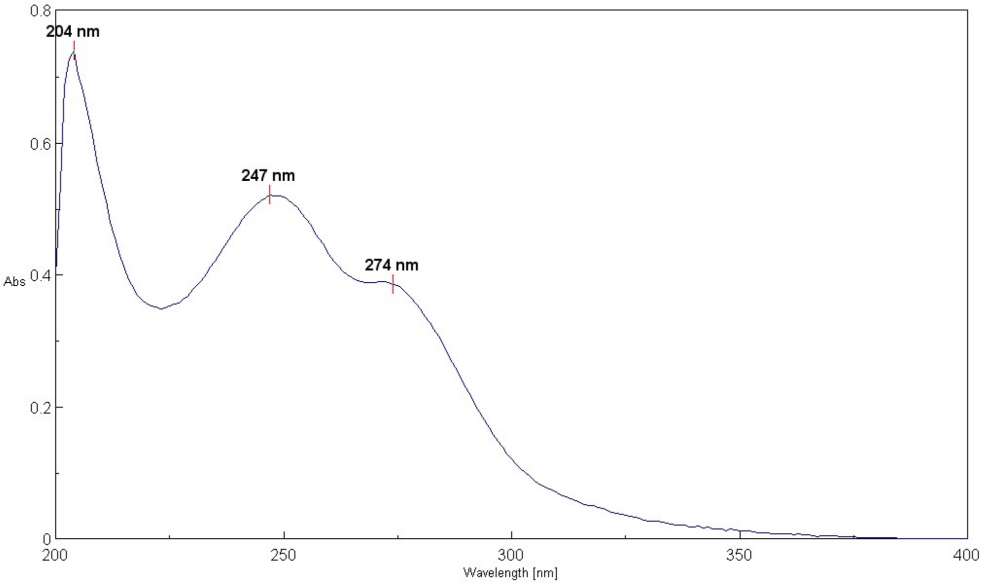

Figure S26 UV spectrum of **3**

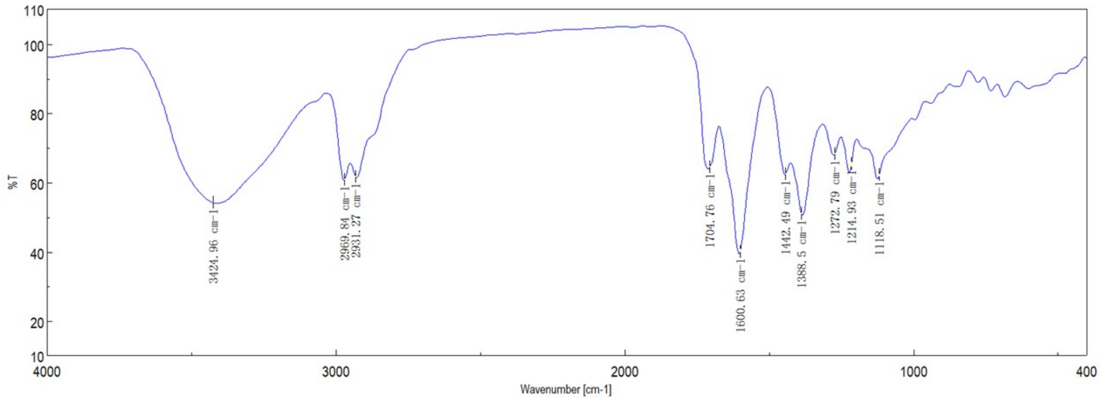

Figure S27 IR spectrum of **3**

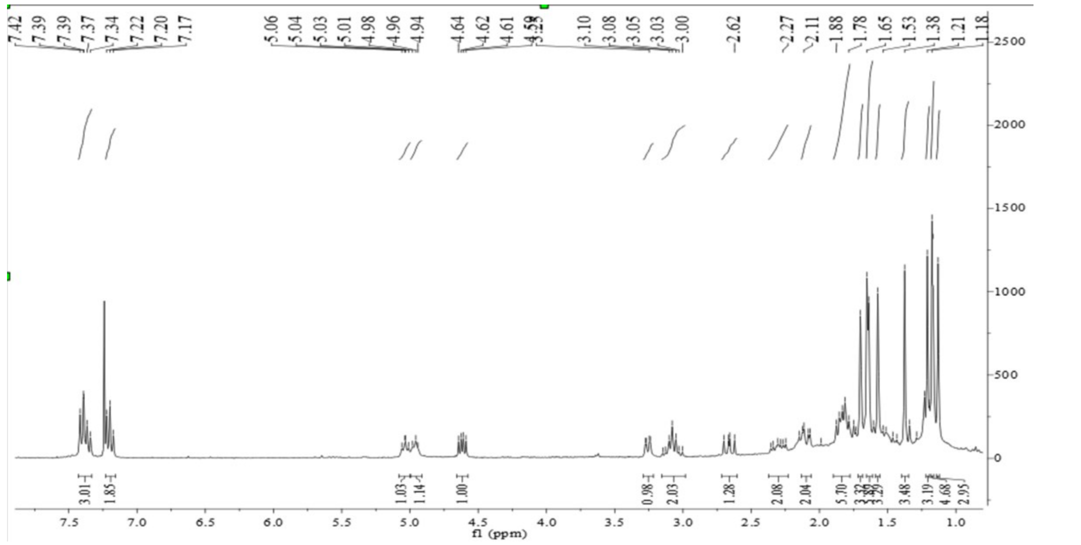

Figure S28 ^{1}H NMR spectrum of **3**

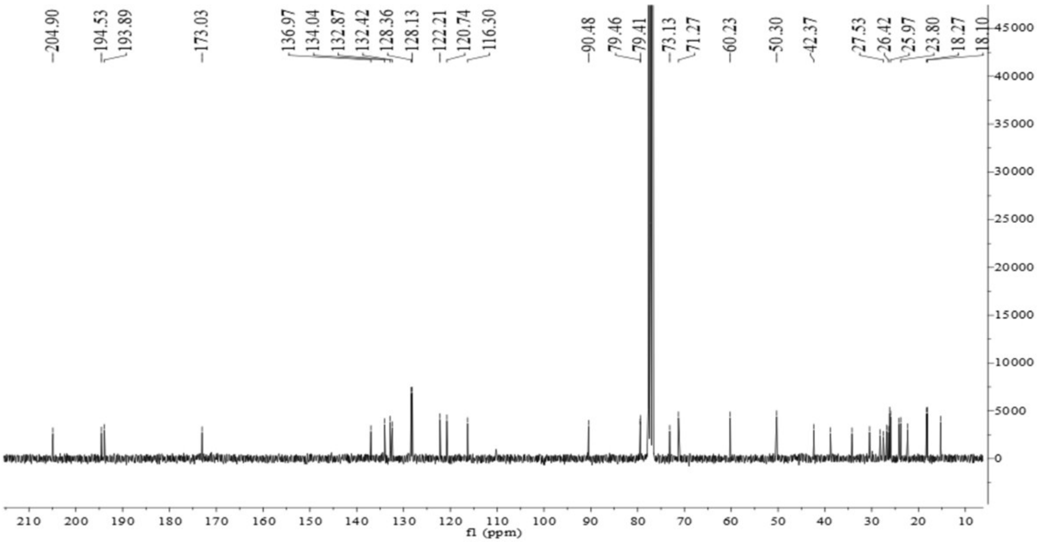

Figure S29 ^{13}C NMR spectrum of **3**

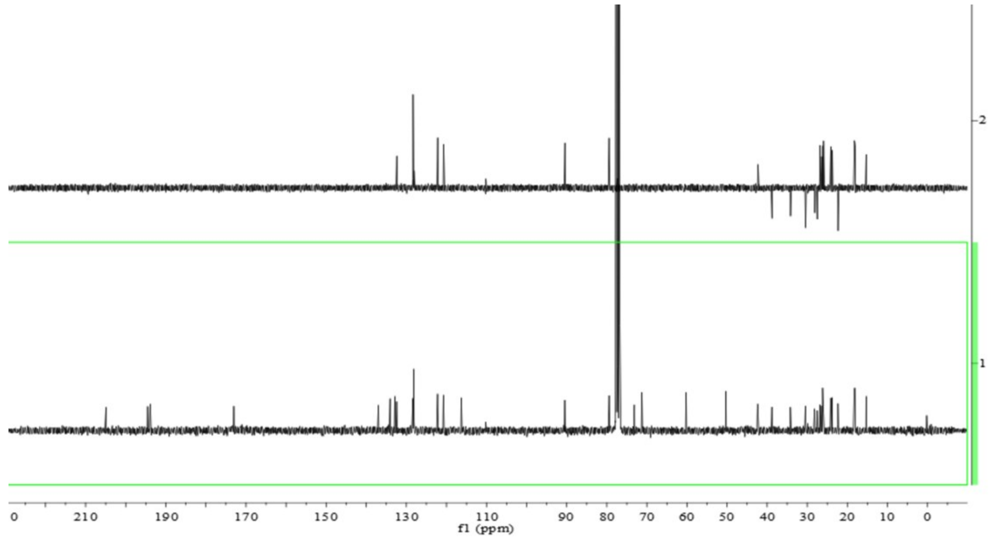

Figure S30 DEPT-135 spectrum of **3**

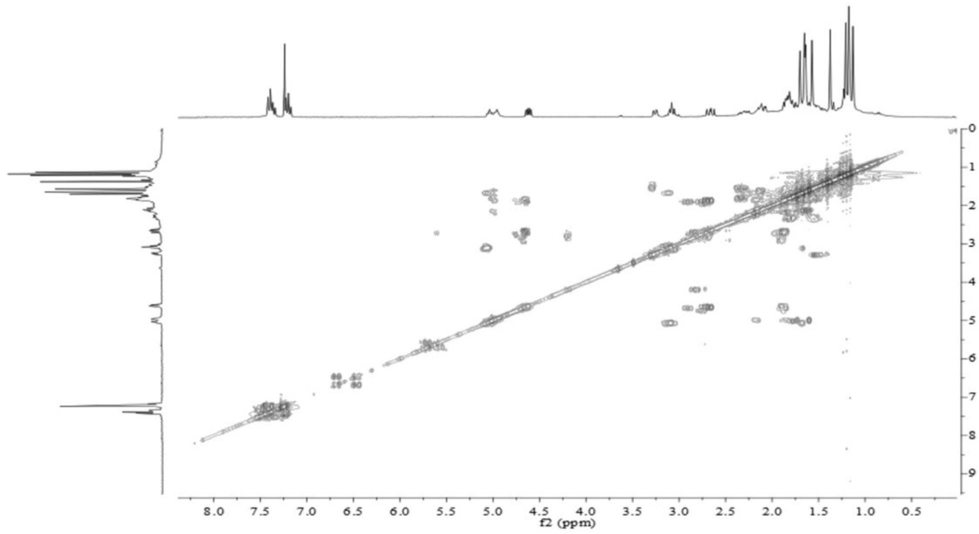

Figure S31 ^{1}H-^{1}H COSY spectrum of **3**

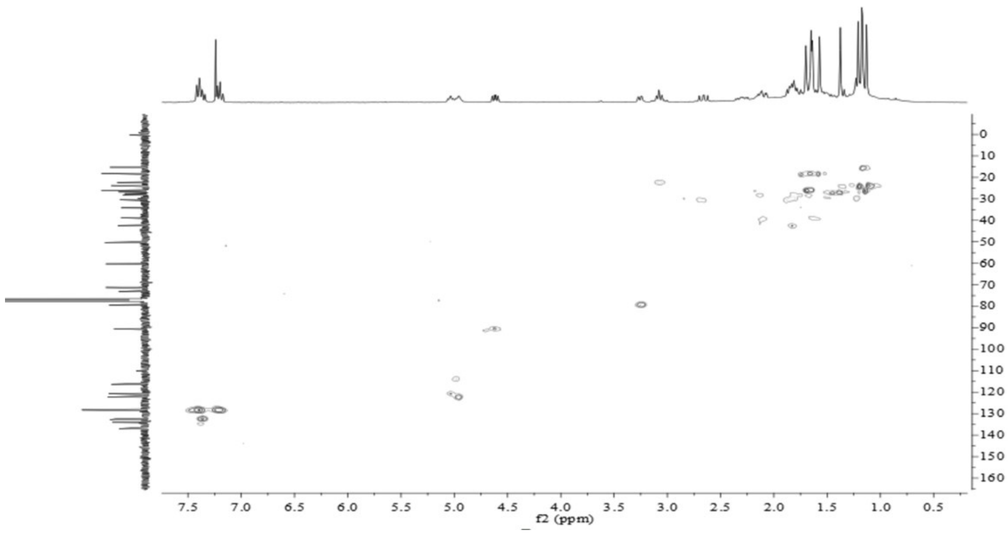

Figure S32 HSQC spectrum of **3**

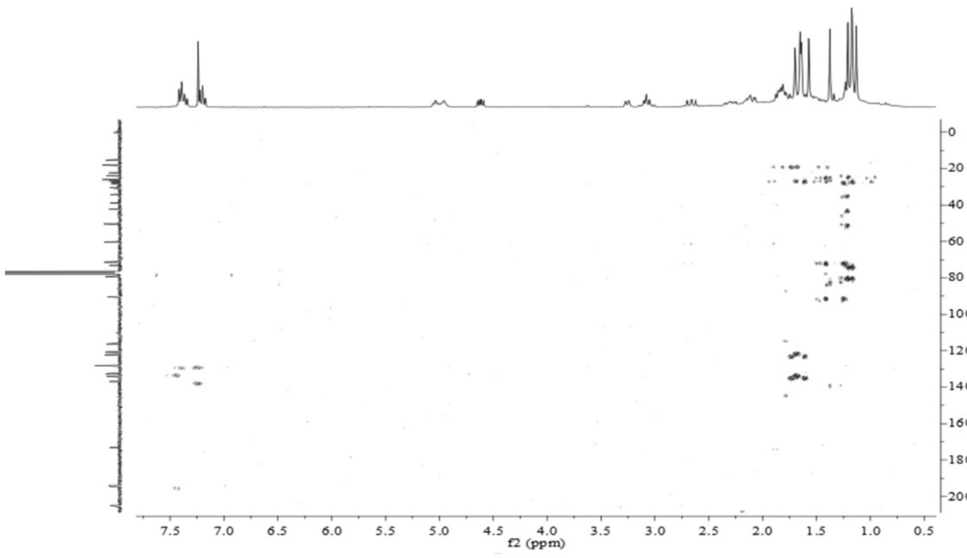

Figure S33 HMBC spectrum of **3**

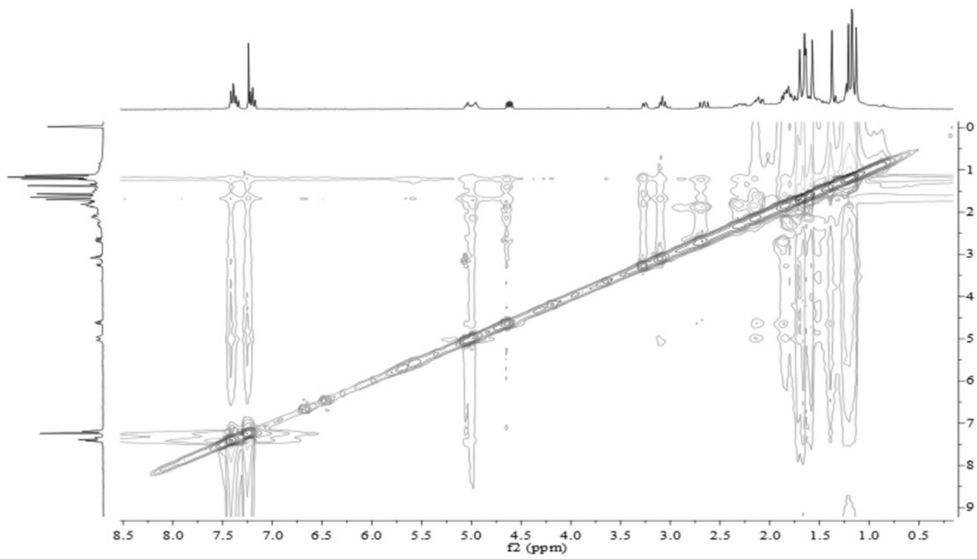

Figure S34 NOESY spectrum of **3**

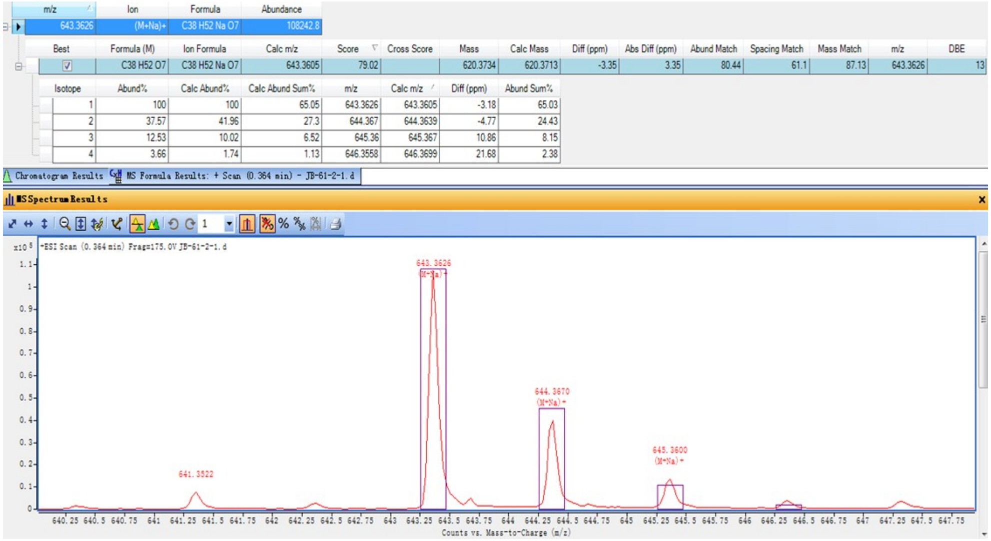

Figure S35 HR-ESI-MS of **4**

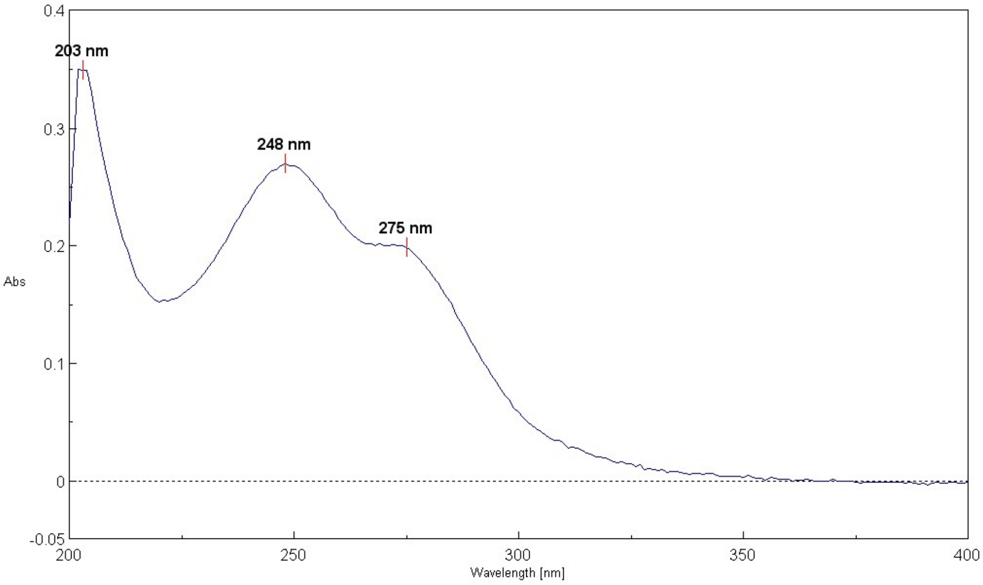

Figure S36 UV spectrum of **4**

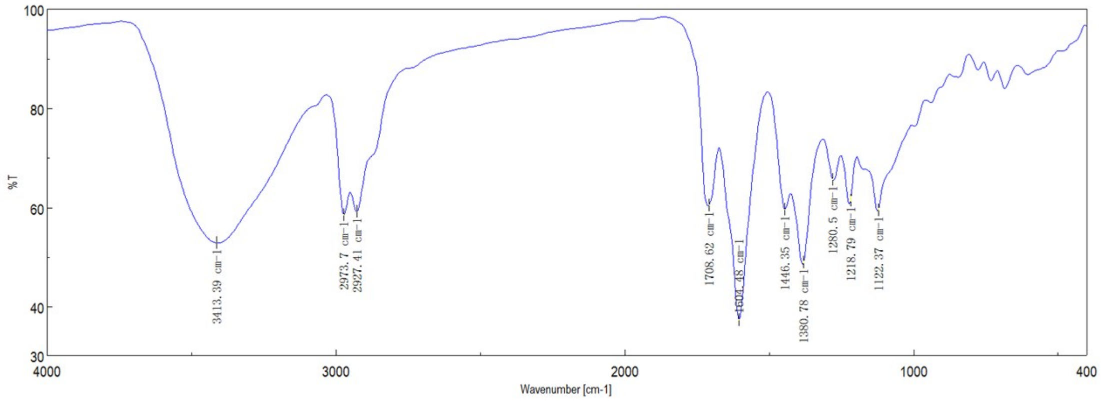

Figure S37 IR spectrum of **4**

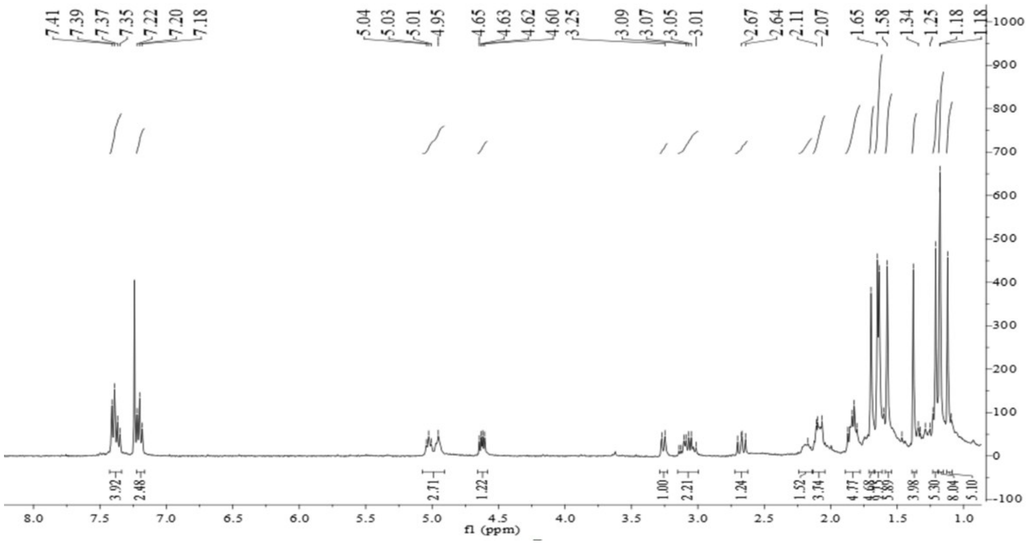

Figure S38 ^{1}H NMR spectrum of **4**

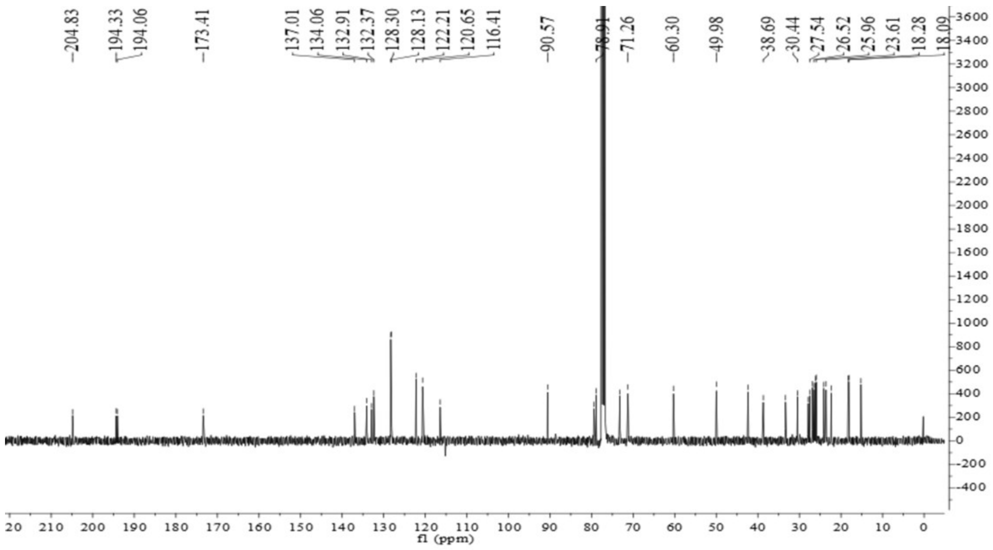

Figure S39 ^{13}C NMR spectrum of **4**

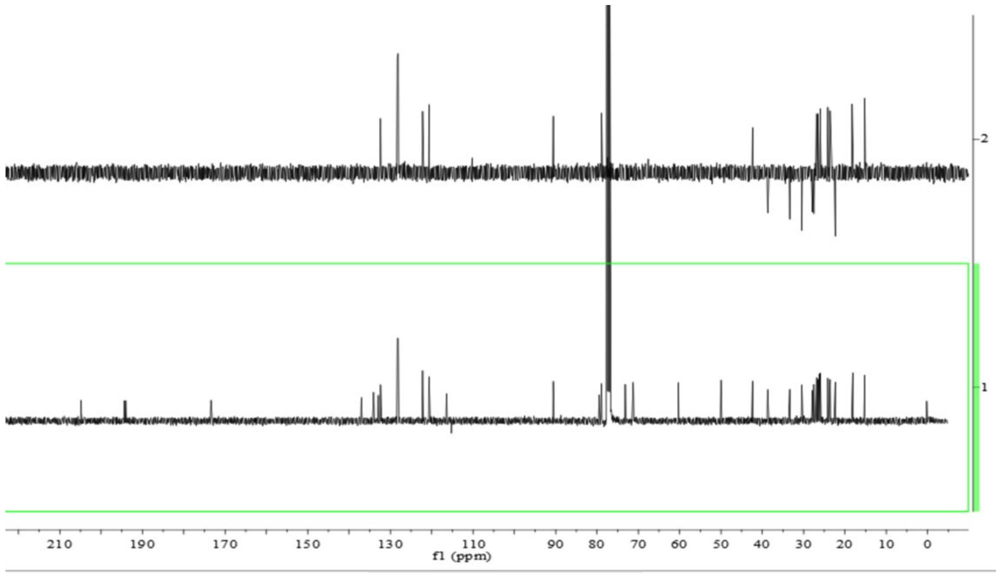

Figure S40 DEPT-135 spectrum of **4**

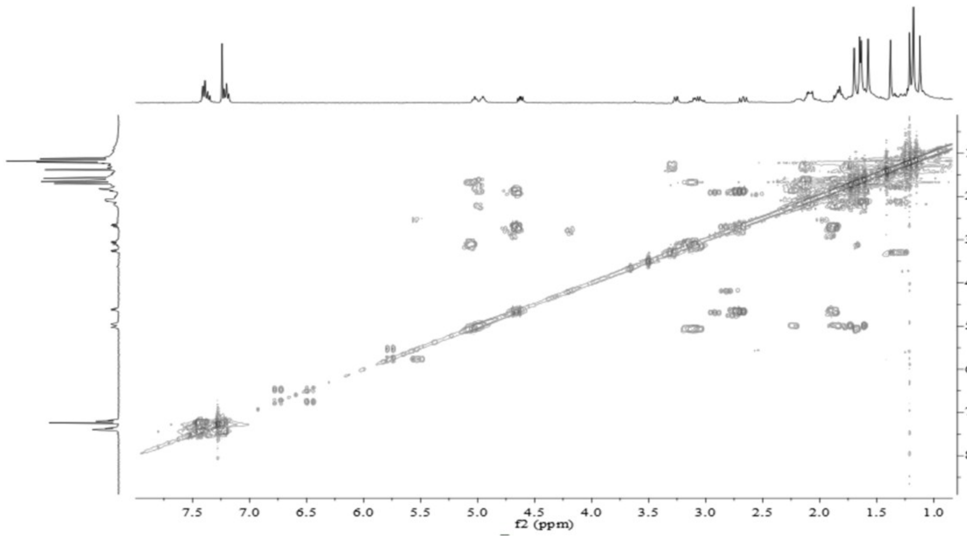

Figure S41 ^{1}H-^{1}H COSY spectrum of **4**

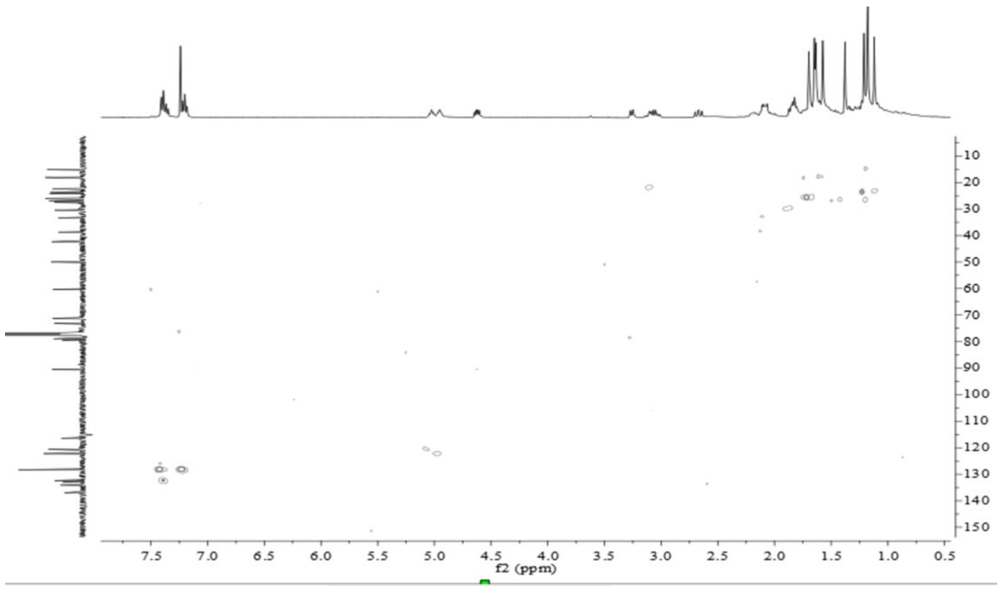

Figure S42 HSQC spectrum of **4**

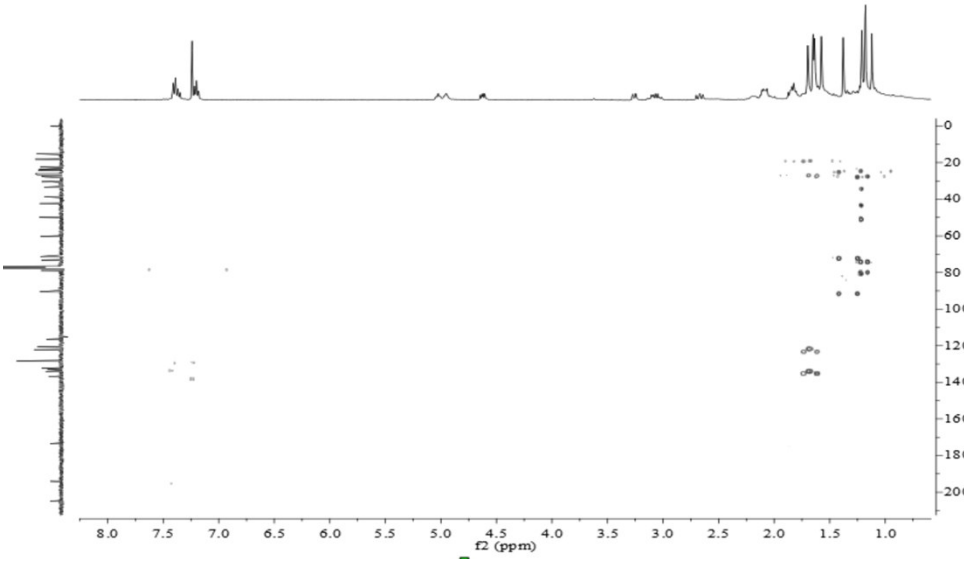

Figure S43 HMBC spectrum of **4**

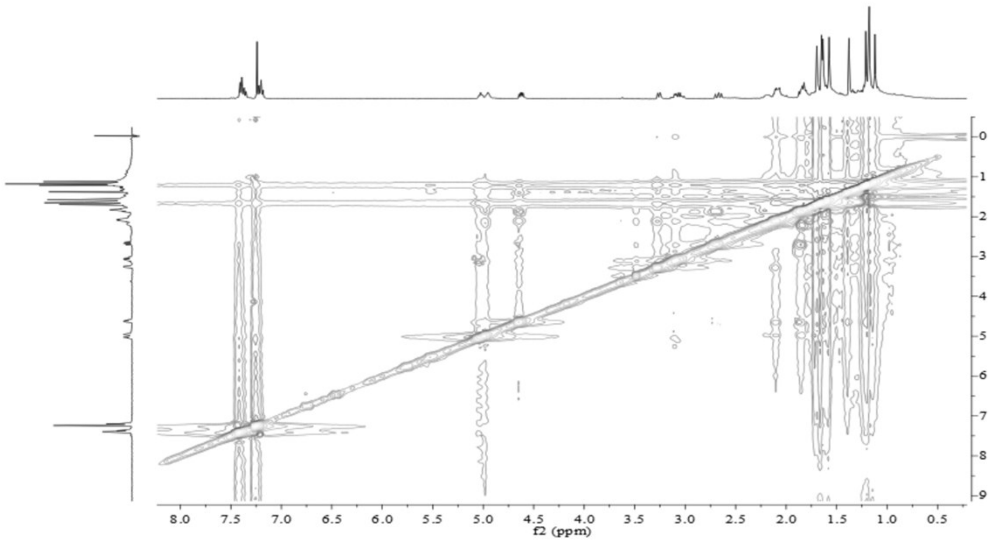

Figure S44 NOESY spectrum of **4**

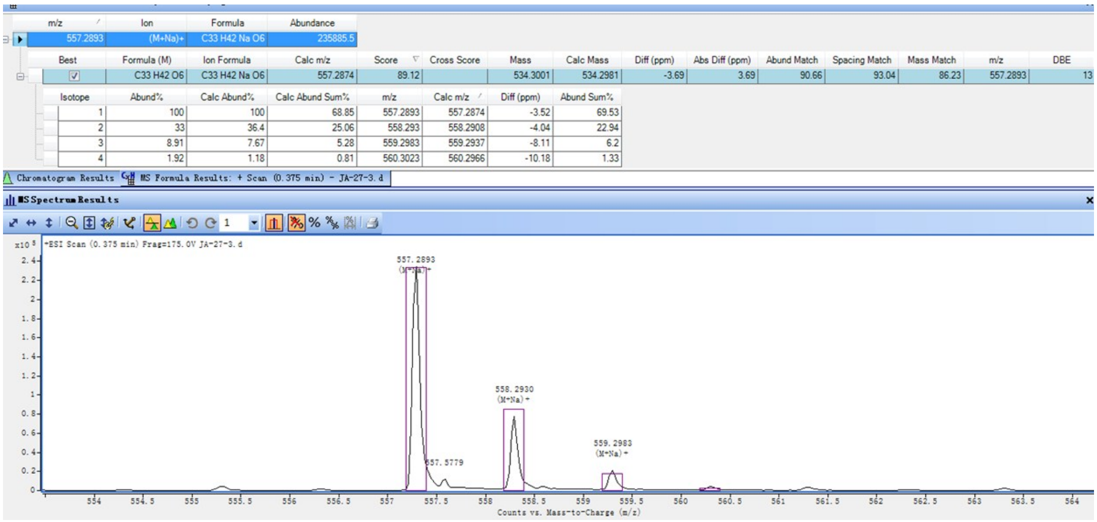

Figure S45 HR-ESI-MS of **5**

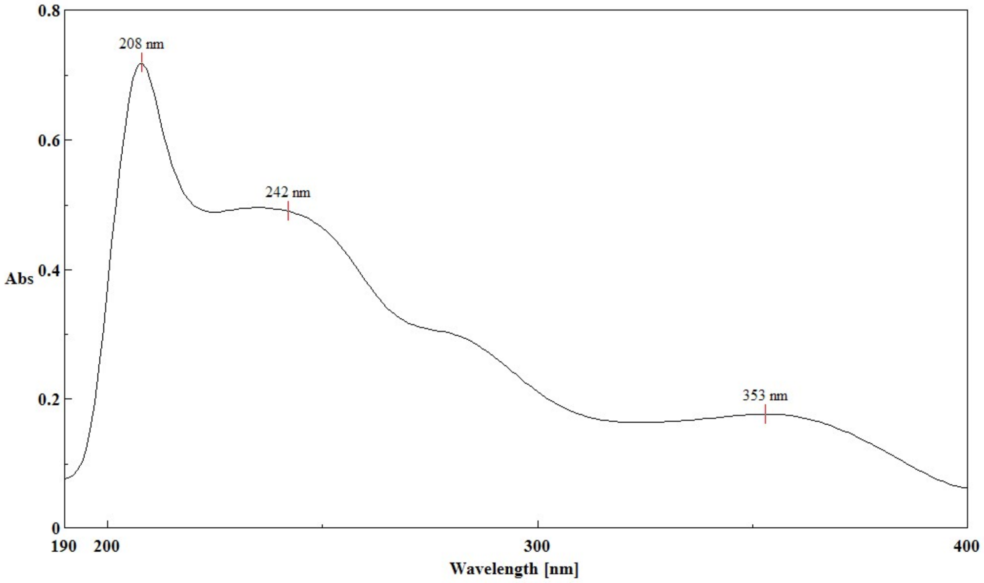

Figure S46 UV spectrum of **5**

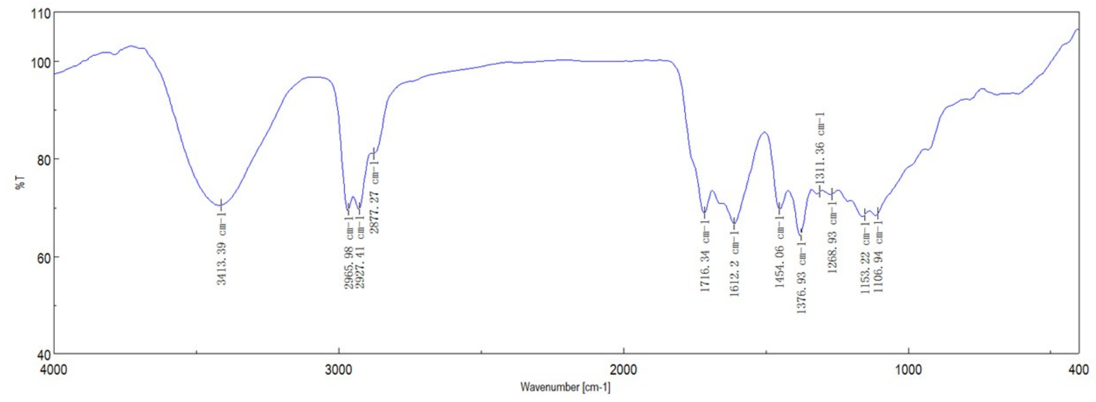

Figure S47 IR spectrum of **5**

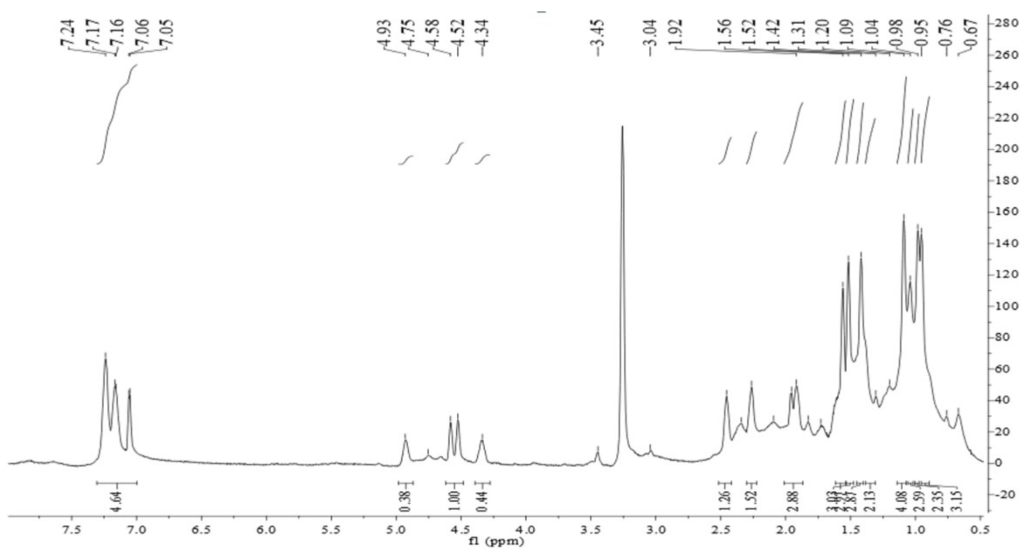

Figure S48 ^{1}H NMR spectrum of **5**

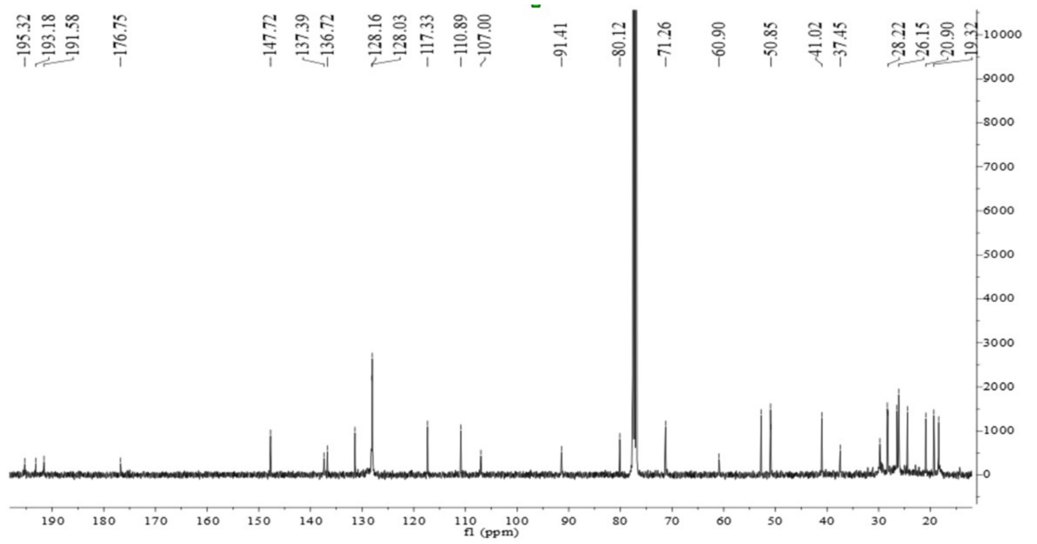

Figure S49 ^{13}C NMR spectrum of **5**

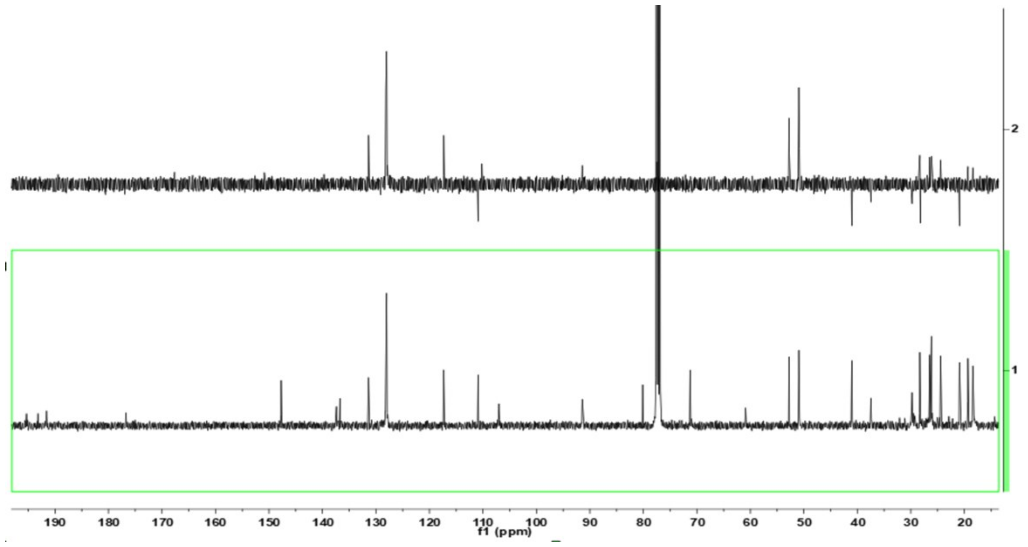

Figure S50 DEPT-135 spectrum of **5**

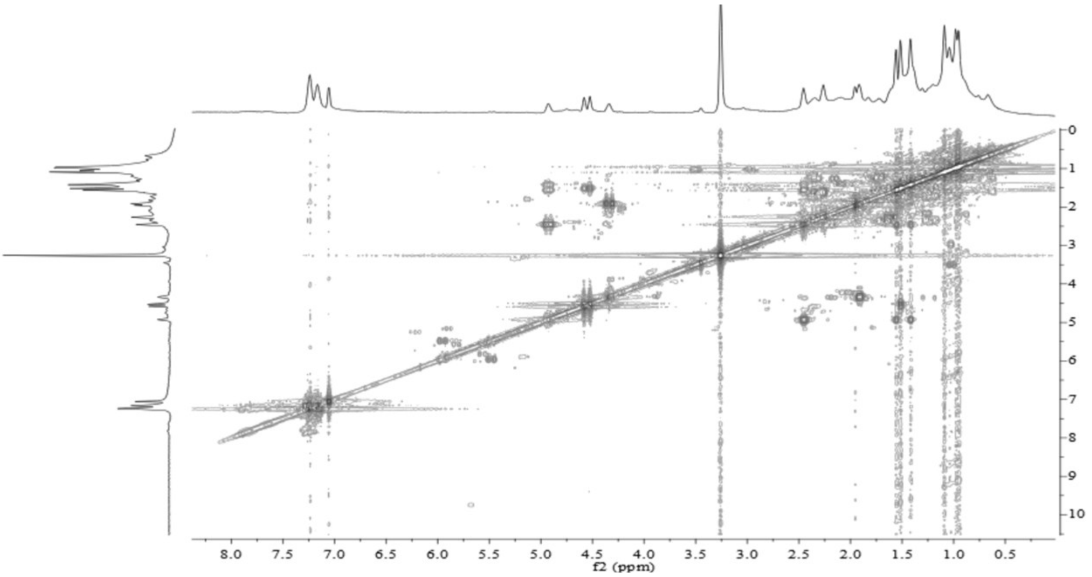

Figure S51 ^{1}H-^{1}H COSY spectrum of **5**

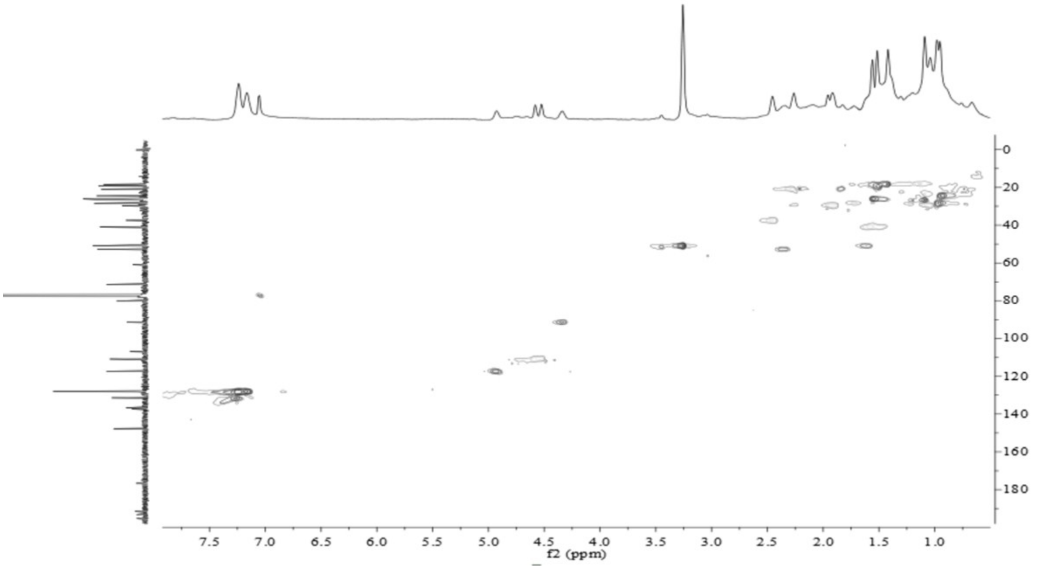

Figure S52 HSQC spectrum of **5**

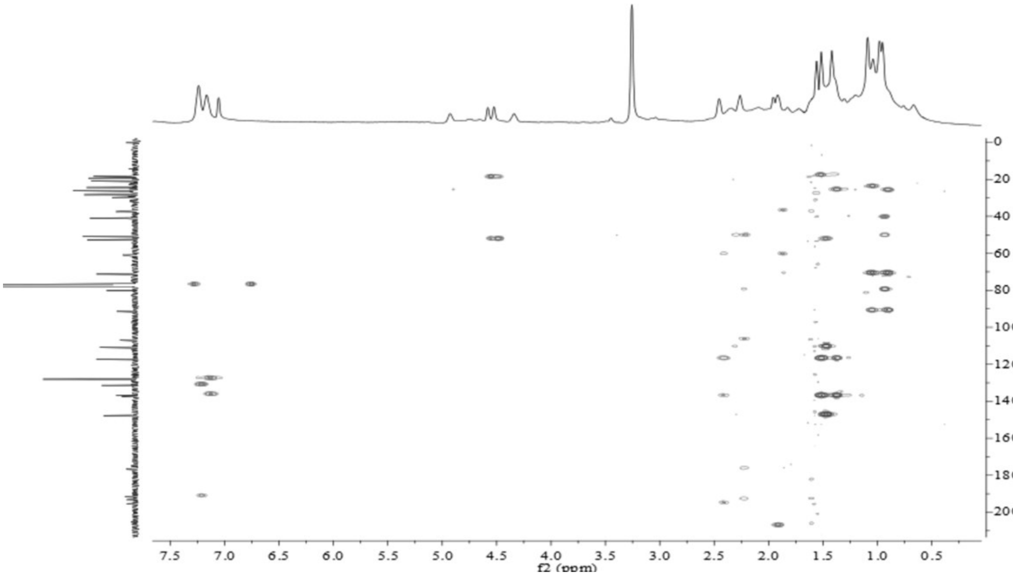

Figure S53 HMBC spectrum of **5**

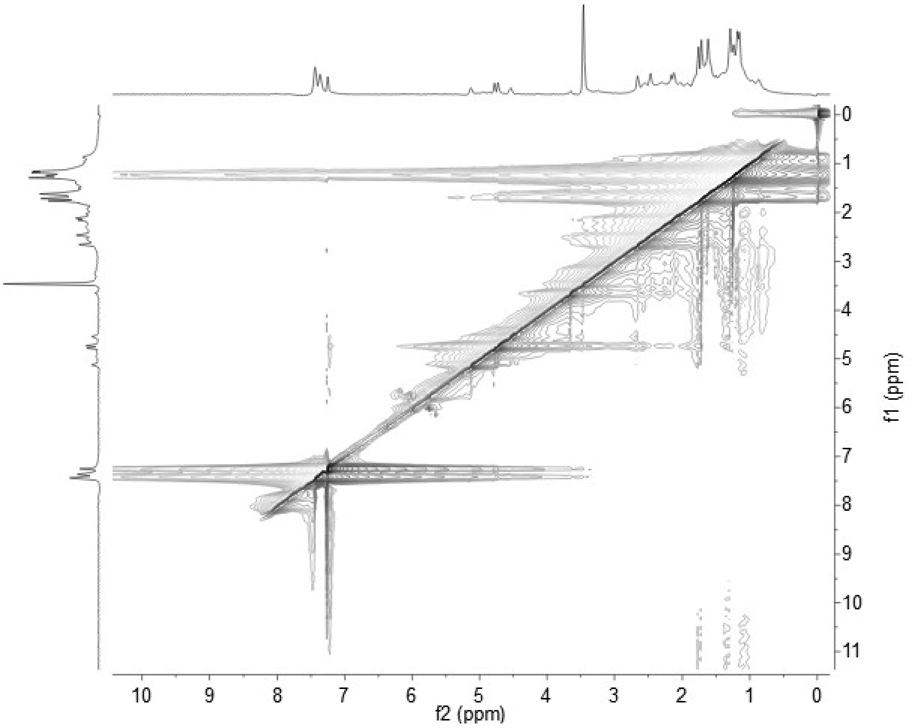

Figure S54 NOESY spectrum of **5**

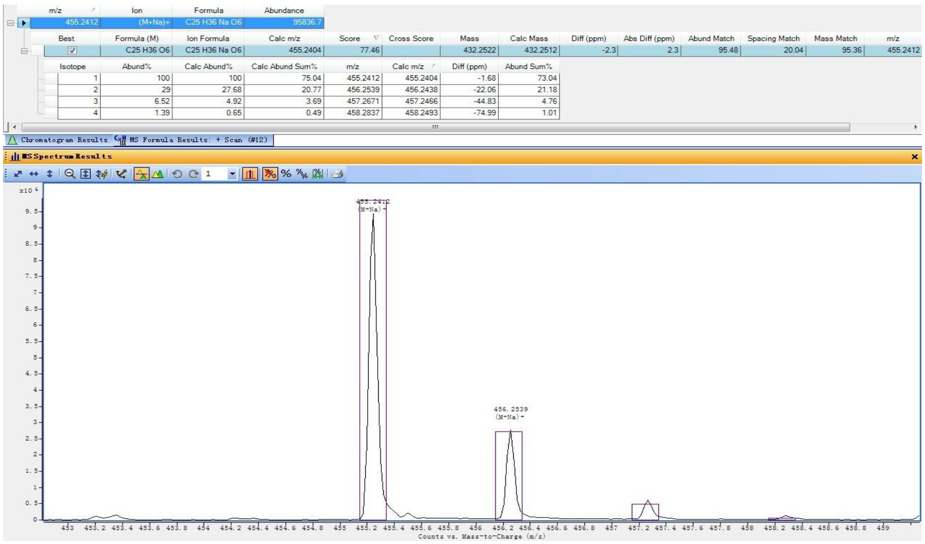

Figure S55 HR-ESI-MS of **6**

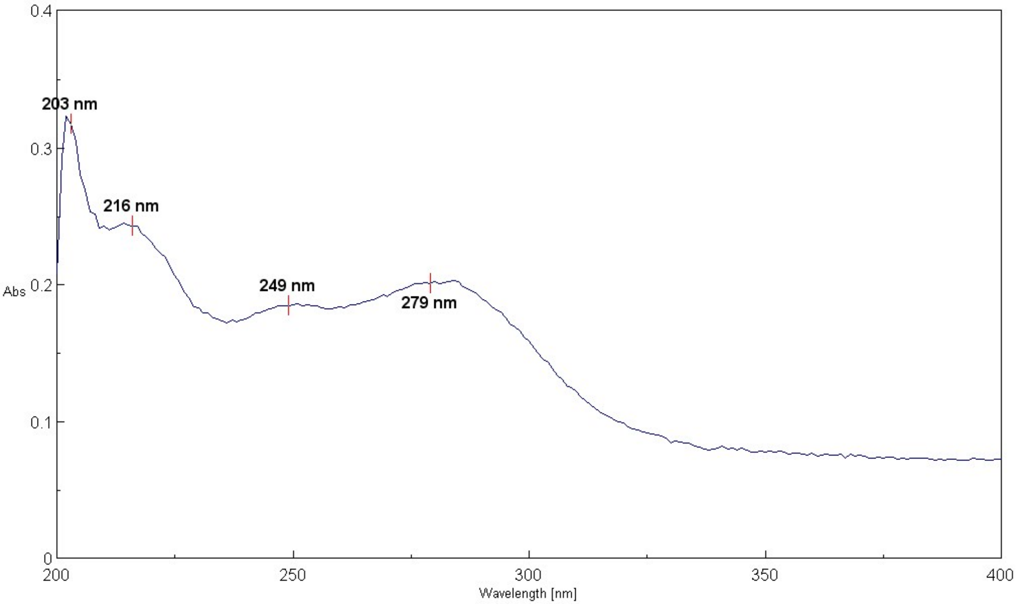

Figure S56 UV spectrum of **6**

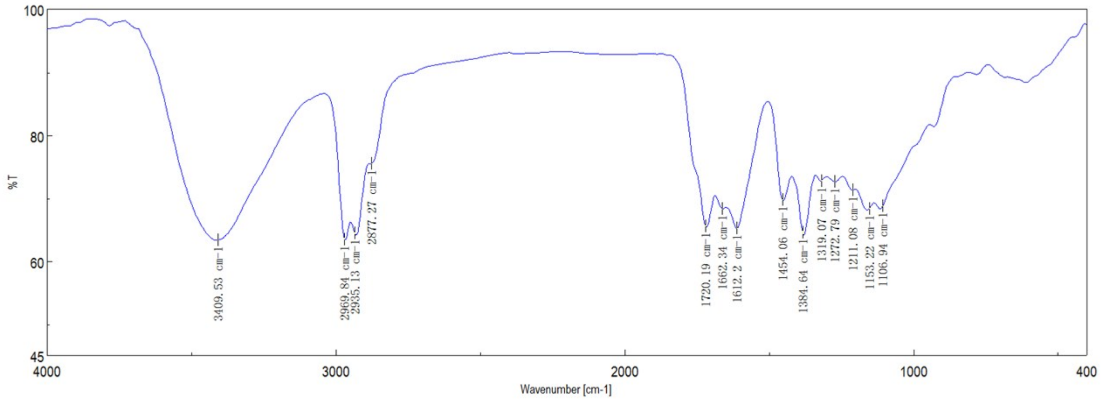

Figure S57 IR spectrum of **6**

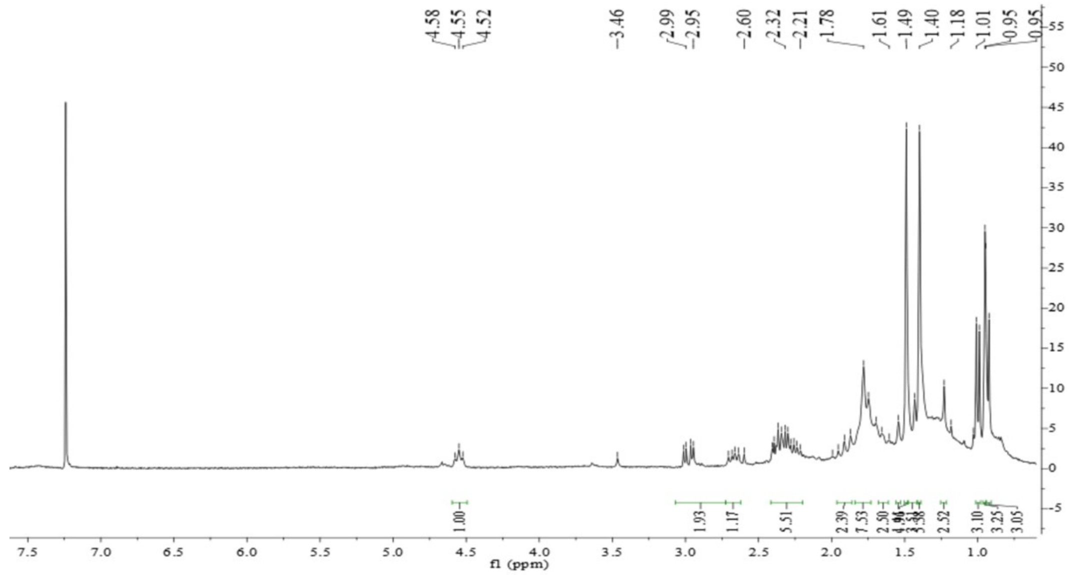

Figure S58 ^{1}H NMR spectrum of **6**

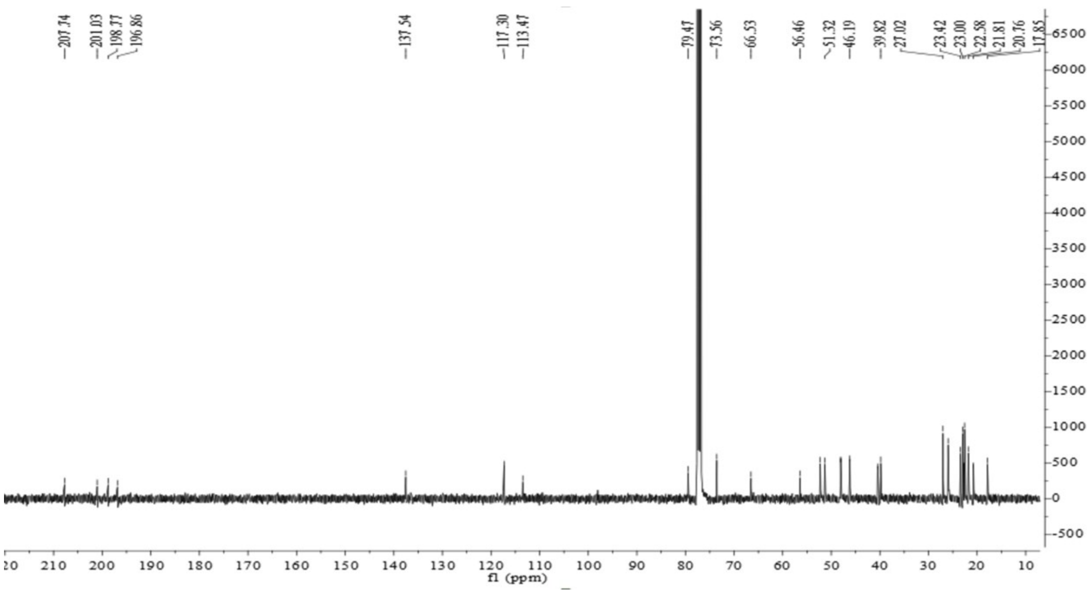

Figure S59 ^{13}C NMR spectrum of **6**

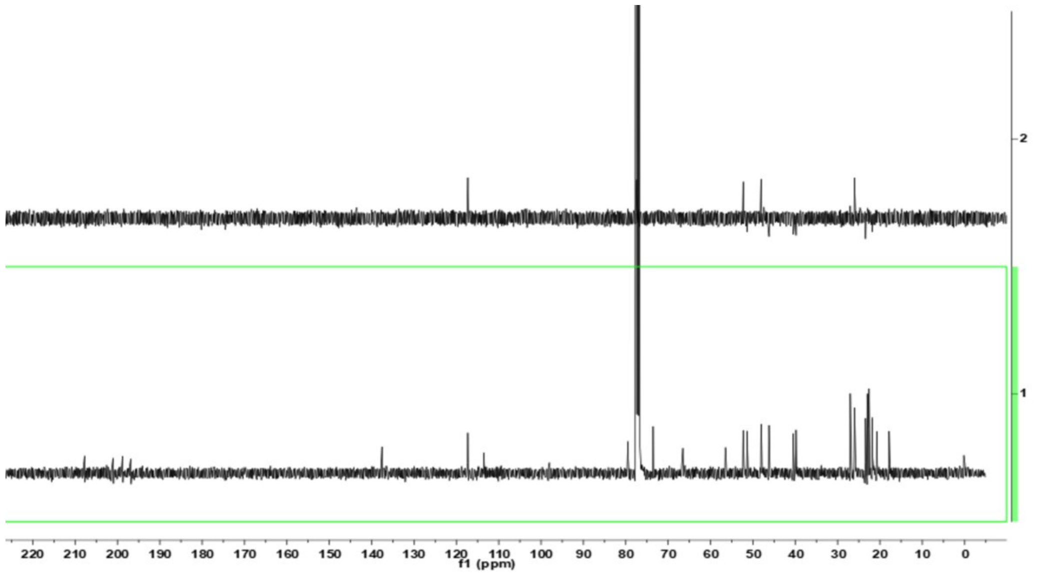

Figure S60 DEPT-135 spectrum of **6**

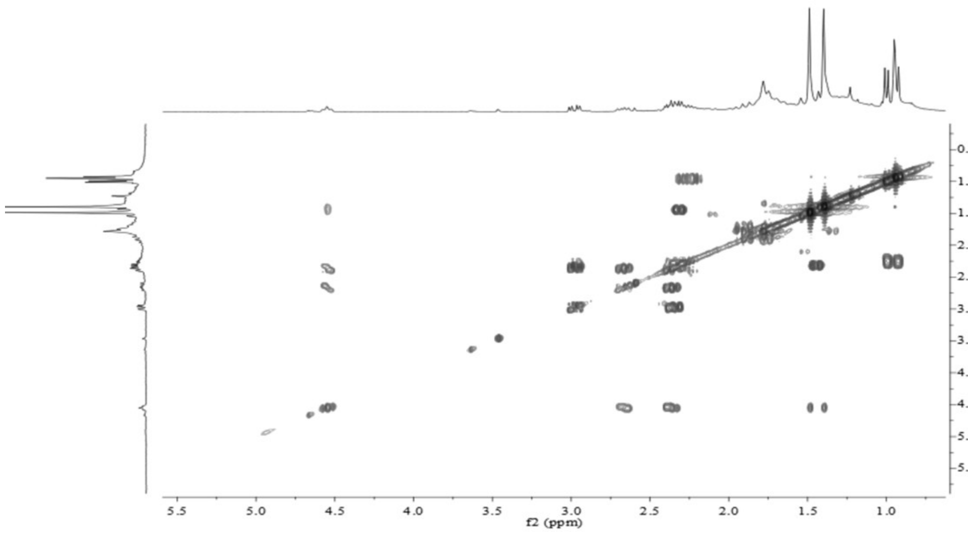

Figure S61 ^{1}H-^{1}H COSY spectrum of **6**

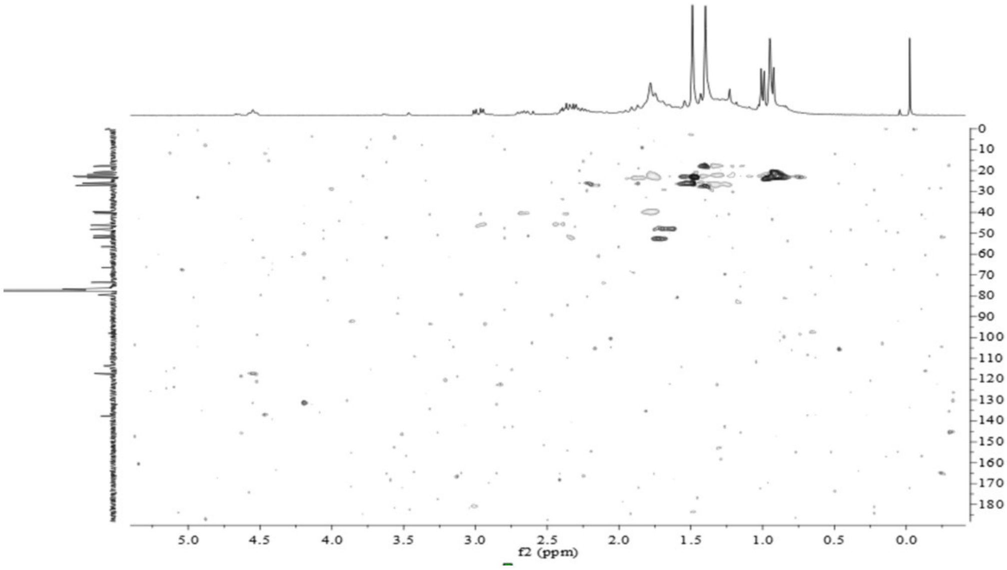

Figure S62 HSQC spectrum of **6**

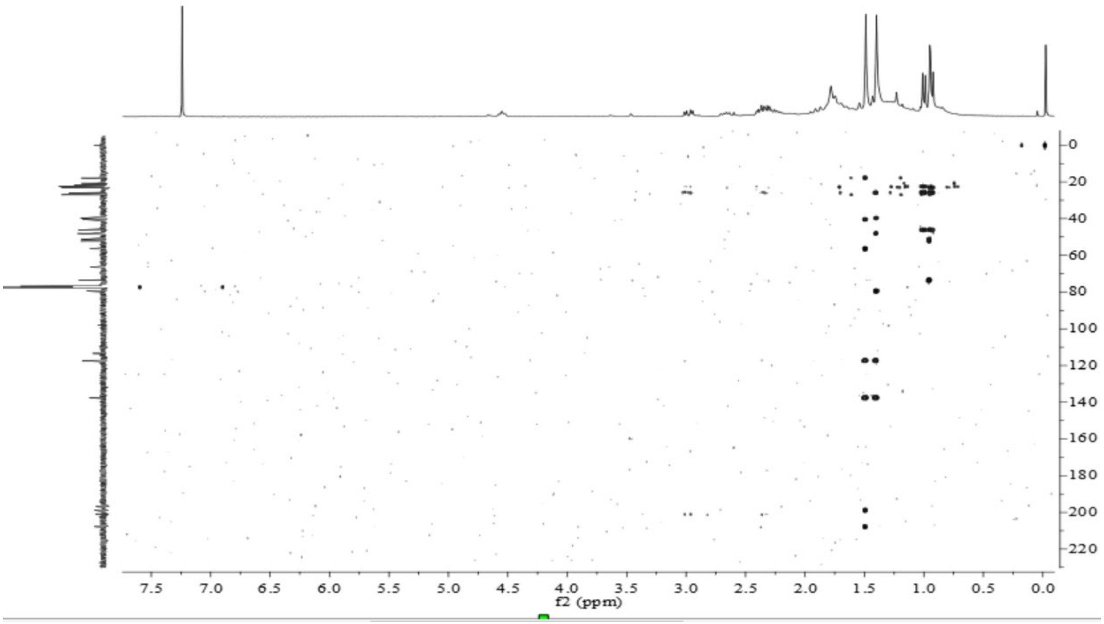

Figure S63 HMBC spectrum of **6**

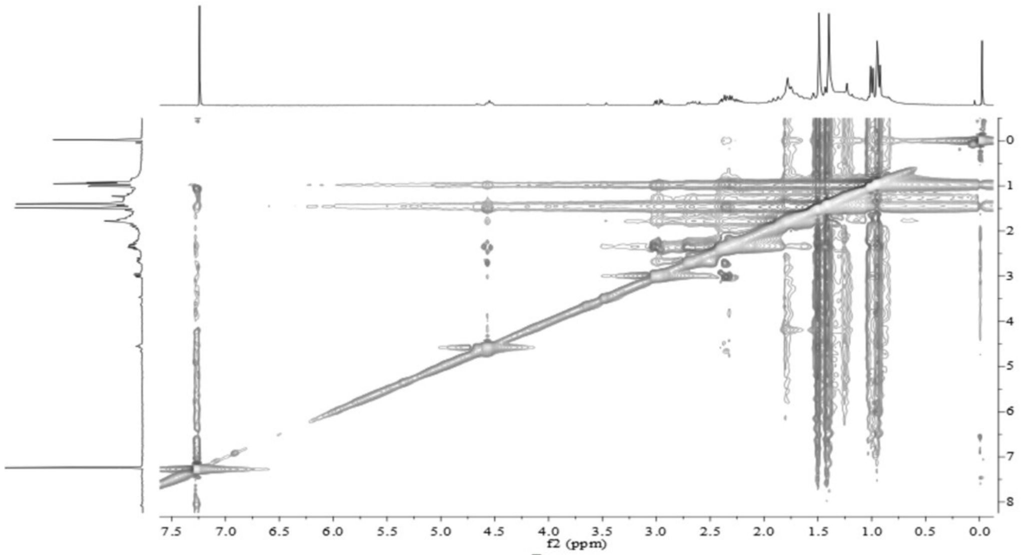

Figure S64 NOESY spectrum of **6**
